# Self-Regulating Hydrogel with Reversible Optical Activity in Its Gel-to-Gel Transformation

**DOI:** 10.1021/jacs.5c03844

**Published:** 2025-05-09

**Authors:** Jingjing Li, Fang Yin, Jianhong Wang, Huachuan Du, Fan Xu, Stefan Meskers, Yudong Li, Stefan Wijker, Yu Peng, Riccardo Bellan, Ghislaine Vantomme, Jian Song, Chun-Sen Liu, E. W. Meijer

**Affiliations:** † College of New Energy, 117776 Zhengzhou University of Light Industry, Zhengzhou 450002, China; ‡ Institute for Complex Molecular Systems and Laboratory of Macromolecular and Organic Chemistry, 3169 Eindhoven University of Technology, Eindhoven 5600 MB, Netherlands; § School of Chemistry and Chemical Engineering, Henan University of Technology, Zhengzhou 450001, China; ∥ School of Chemical Engineering and Technology, 12605 Tianjin University, Tianjin 300350, China

## Abstract

This study reports a supramolecular gel system capable of dynamic gel-to-gel transformations and reversible inversion of optical activity between superhelical and single-helical structures without passing through a sol phase. Inspired by collagen-like adaptability, the system utilizes 4-pyridinylboronic acid and guanosine as building blocks. Hierarchical assembly is achieved through pH-responsive boronic ester formation and guanosine-mediated G-quadruplex stacking, enabling transitions between superhelices and single helices with opposite optical activity. The system employs three regulatory pathways: bidirectional pH modulation, monotonic pH increase, and monotonic pH decrease, demonstrating programmable and reversible control over chirality, morphology, and mechanical properties. In the autonomous pH regulation, we have created an out-of-equilibrium hydrogel system with controlled switching of optical activity. Unlike traditional gel–sol–gel systems, this gel maintains macroscopic stability during transformations. Our remarkable finding bridges the gap between static supramolecular assemblies and dynamic soft materials, offering a platform for designing functional, biomimetic systems. The combination of hierarchical organization, dynamic chirality control, and robust programmability positions this gel for applications in adaptive optics, responsive biomaterials, and programmable soft matter.

## Graphical abstract

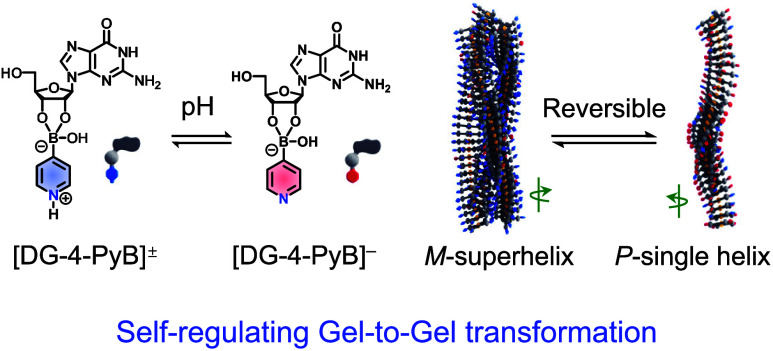

## Introduction

Superhelical architectures are ubiquitous in nature, playing crucial roles in the stability, adaptability, and functionality of biological systems.^1,2^ These complex structures typically emerge from the hierarchical assembly of simpler helical units, with sometimes the opposite chirality. For example, collagen, a key structural protein, achieves a right-handed supercoil by intertwining three left-handed α-chains (Scheme 1).^3^ This hierarchical organization, coupled with dynamic conformational adaptability, enables collagen to provide structural support and mediate diverse biological processes.^4^ Translating such hierarchical complexity and dynamic inversion of optical activity behavior into synthetic systems remains a formidable challenge in materials science.

**Scheme 1:**
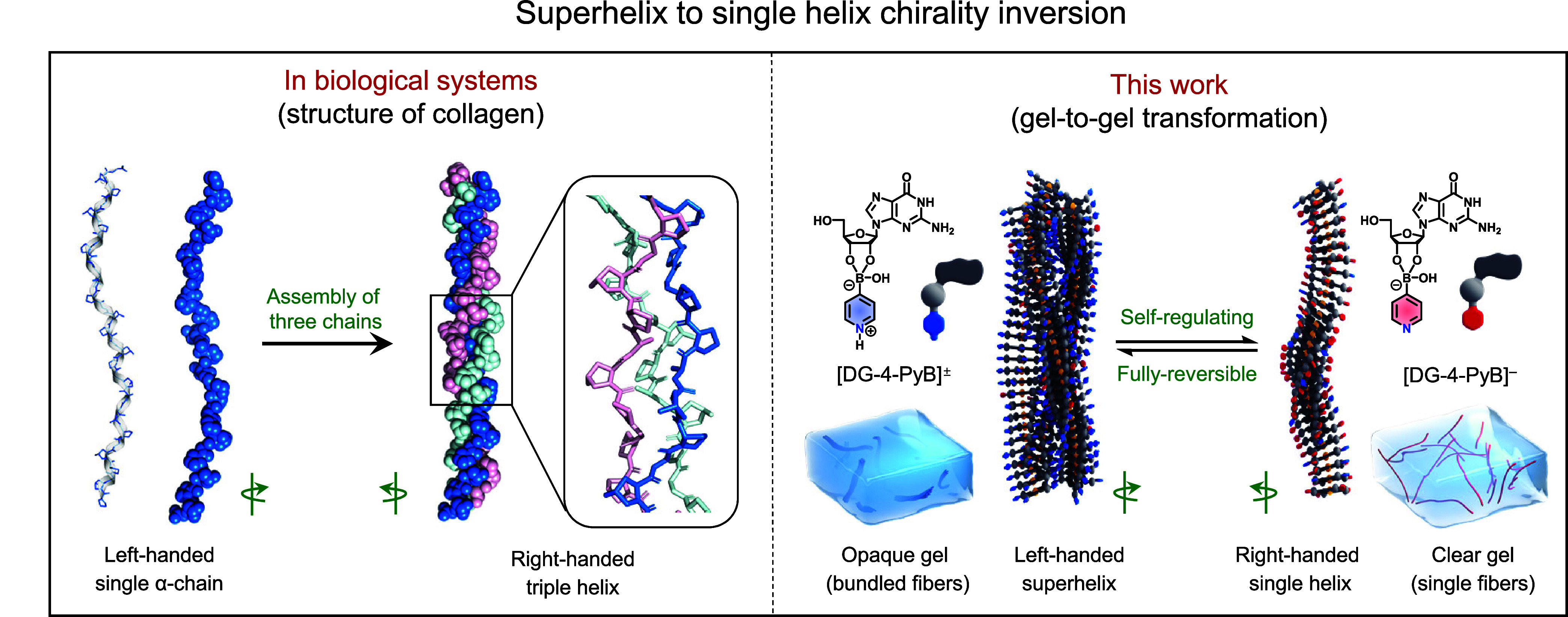
Chirality Inversion between Superhelix and Single Helix in Collagen and Its Comparison with an Artificial Supramolecular System^a^

a The crystal structure of collagen was derived from PDB ID 1CGD.

Supramolecular assembly, driven by reversible noncovalent interactions, has emerged as a versatile and robust strategy for constructing complex hierarchical structures with tunable chirality.^5–7^ Over the past decades, substantial progress has been achieved in controlling supramolecular chirality in artificial systems through rational molecular design,^8–11^ or the application of external stimuli.^12–16^ Our group has discovered that assembly pathways in supramolecular polymer systems during the nucleation–elongation process critically influence the helical handedness of supramolecular polymers.^17,18^ These findings expand the understanding of chirality control mechanisms and suggest a new direction for exploring pathway-dependent chirality regulation in supramolecular systems.^19–21^ Moreover, supramolecular chirality inversion in these systems has unlocked various functionalities, including chiral recognition, sensing, chiroptical switching, enantiomer separation, asymmetric catalysis, circularly polarized luminescence, chiral-induced spin selectivity, and chiral bioeffects.^22,23^ However, current systems primarily focus on reversals of optical activity or morphological transformations in static assemblies.^22^ The dynamic, multiscale control over structure and chirality remains largely unexplored, with only a few examples demonstrated in solution-phase assemblies.^24–26^ These solution systems benefit from unrestricted molecular dynamics, enabling facile manipulation of supramolecular structures.^27^ However, they often suffer from poor stability and mechanical robustness, limiting their practical applications, especially where stability and strength are essential.

Hydrogels, as typical semisolid soft materials, are the most relevant biochemical scaffold due to their tunable properties, inherent biocompatibility, and similarity with tissue and cell environments.^28–30^ The introduction of chirality further increases the complexity of hierarchical structures and extends the functionality of gel materials toward various advanced applications.^23,31^ Among recent developments, gel-to-gel transformations stand out as an intriguing avenue, offering opportunities to create functional materials through structural reconfigurations.^32,33^ However, most gel-to-gel transitions reported thus far rely on intermediate solution phases (Gel–Sol–Gel) or external stimuli, such as temperature, light, or chemical inputs, to induce transformations and chirality inversion.^32,34–36^ These constraints limit their applicability in continuous, adaptable environments. Due to restricted molecular motion within the three-dimensional (3D) solid fiber network,^27^ it remains highly challenging in gel systems to achieve continuous dynamic inversion of optical activity, particularly in an autonomous and adaptive manner akin to natural systems. Addressing these challenges requires innovative approaches that integrate dynamic control, hierarchical organization, and macroscopic stability.

In this study, we present a novel supramolecular gel system capable of direct gel-to-gel transformations coupled with the reversible inversion of optical activity between superhelix and single helix configurations (Scheme 1). The system utilizes the unique properties of 4-pyridinylboronic acid (4-PyB), which adopts an sp^3^ boron configuration under neutral conditions (Figure S1),^37^ and guanosine as complementary building blocks.^38–40^ By incorporating biocatalytic reaction networks to autonomously regulate pH, the resulting gels exhibit exceptional robustness, maintaining a continuous gel network during structural reconfigurations while enabling dynamic adaptability and precise control over chirality, morphology, and mechanical properties. By combining hierarchical assembly, dynamic chirality inversion, and self-regulation, this system sets a new benchmark for programmable, biomimetic chiral soft materials with broad potential in adaptive optics, responsive biomaterials, and beyond.

## Results and Discussion

### Properties and Assembly Mechanisms of Supramolecular Hydrogels

The interaction between boronic acids and the cis-diol of guanosine to form boronic esters is well-documented.^41–43^ Under suitable pH conditions, mixing natural d-guanosine (DG) with 4-PyB produces the boronic ester [DG-4-PyB]**^–^** (Figure 1a). With the right temperature and concentration, these boronic esters form supramolecular hydrogels. Computational analysis (SI for details) reveals p*K*_a_ values of 8.4 and 11.3 for the pyridinyl nitrogen (N) and (N7)H sites of [DG-4-PyB]^−^, respectively (Figure 1a). The speciation of [DG-4-PyB]^−^ evolves dynamically with pH, giving rise to distinct phase transitions from precipitate to gel to solution (Figure 1b–l). The gels formed exhibit pH-responsive inversions of the optical activity and morphological variations (Figure 1h–p). At pH ∼ 3.0, mixing DG (1.5%, w/v), 4-PyB, and KCl in a 1:1:0.5 ratio results in precipitation (Figure 1c,h). ^11^B NMR confirms the absence of boronic ester formation under these conditions, with only the sp^2^ B signal observed at approximately 16 ppm. (Figure S2). Scanning electron microscopy (SEM) images reveal discrete particulate morphologies (Figure 1h). At pH ∼ 5.0 and above, ^11^B NMR spectra confirm that mixing DG and 4-PyB results in boronic ester formation, with a new sp^3^ boron signal appearing between 7–8 ppm (Figure S2). ^1^H NMR spectra and mass spectrometry further support the pH-dependent evolution of the chemical speciation of the boronic ester (Figures S3–S4). Specifically, at pH ∼ 5.0, the predominant species is the protonated boronic ester [DG-4-PyB]^±^, which assembles to form a clear hydrogel (Figure 1d,i). Cryo-TEM reveals fiber assemblies with some individual fibers merging or intertwining with each other, forming a multifiber structure (Figure 1i). Circular Dichroism (CD) spectra exhibit a positive signal centered at ∼300 nm, indicating helical chirality (Figure 1n). At pH ∼ 7.5, [DG-4-PyB]^±^ and [DG-4-PyB]^−^ coexist, forming an opaque hydrogel (Figure 1e,j). Cryo-TEM and SEM reveal left-handed helical fiber bundles with diameters up to 50 nm formed by the intertwining of single fibers (Figure 1j,m). CD spectra display a pronounced negative band centered at ∼305 nm, assumed to be in line with the left-handed *M*-superhelices (Figure 1o). At pH ∼ 10.0, deprotonated [DG-4-PyB]^2–^ appears alongside [DG-4-PyB]^−^, forming a clear gel (Figure 1f,k). Cryo-TEM images show single fibers with diameters of ∼3.5 nm (Figure 1k), closely matching the theoretical diameter of a G-quartet formed by [DG-4-PyB]^−^ (2.51 nm) (Figure S8). CD spectra show a positive band at 290–315 nm, indicating a *P*-helical chirality (Figure 1p). Although [DG-4-PyB]^2–^ appears at this pH (resulting from deprotonation at the N_1_H site), it cannot assemble, as it lacks the ability to form G-quartet stacking blocks. Therefore, single fibers are believed to be formed solely by [DG-4-PyB]^−^. At pH ∼ 13.5, only [DG-4-PyB]^2–^ is present, precluding assembly and resulting in a homogeneous solution (Figure 1g,l). Rheological analysis reveals that the opaque hydrogel exhibits significantly higher mechanical strength compared to the transparent hydrogel at the same concentration, consistent with their respective fiber bundles and single-fiber structures (Figures 1q and S5).

**Figure 1:**
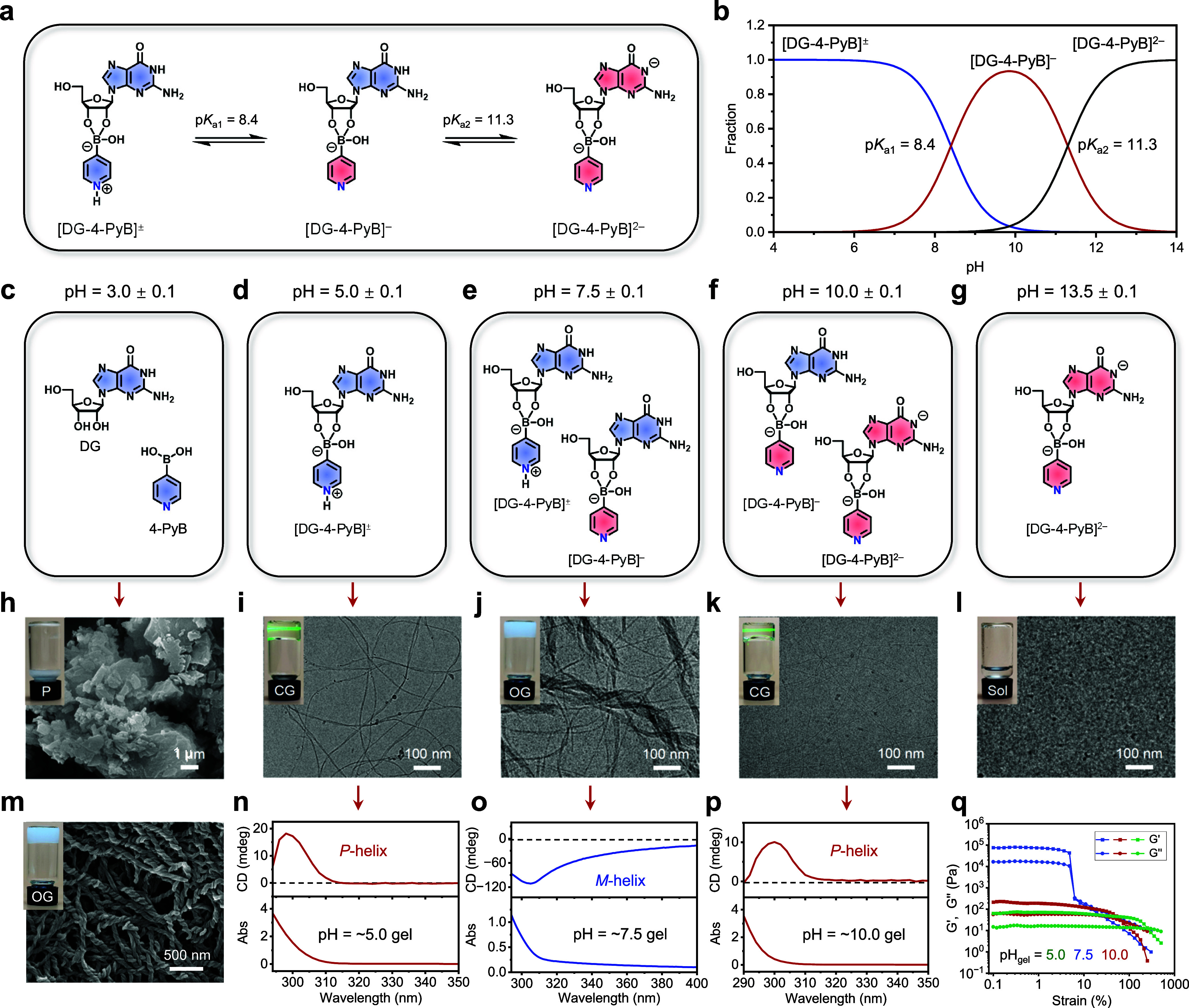
Properties of supramolecular hydrogels. (a, b) Chemical structures (a) and distribution curve (b) of [DG-4-PyB]^−^ species. (c–l) pH-dependent evolution of chemical speciation (c–g) and morphology (h–l). Inset in (h–l): Phase behavior of DG/4-PyB (1:1) mixture in aqueous KOH or HCl under various pH conditions. 0.5 equiv of KCl was added when necessary. (m) SEM image of the opaque gel at pH ∼ 7.5. Inset in m: Photograph of the opaque gel. (n–p) pH-dependent CD and absorption spectra of the gels formed by the DG/4-PyB (1:1) mixture. (q) Rheological dynamic strain sweeps of the gels at a concentration of 2% (w/v) in DG (71 mM). All other concentrations are 1.5% w/v in DG (53 mM), except for the opaque gels, which have a concentration of 1.0% w/v (35 mM) in 1 (j, m), and 0.6% w/v (21 mM) in (o). Optimal sample concentrations have been chosen according to the specific requirements and principles of each characterization method. Abbreviations: P - Precipitate; CG - Clear Gel; OG - Opaque Gel; Sol - Solution.

These results reveal a pH-responsive inversion of the optical activity and morphological changes between single fibers and fiber bundles. Notably, the optical activity of the gels is directly dictated by the intrinsic chirality of guanosine. When DG is replaced by its enantiomer l-guanosine (LG), the optical activity inversion follows an opposite trend: the CD signals show *M*-helical (negative signal) at pH ∼ 5.0, *P*-superhelical (positive signal) at pH ∼ 7.5, and *M*-helical at pH ∼ 10.0 (Figure S6). Cryo-TEM and SEM analyses confirm that the opaque hydrogel formed at pH ∼ 7.5 with LG/4-PyB exhibits right-handed *P*-superhelical bundles (Figure S7), contrasting the left-handed bundles formed with DG/4-PyB. In a 1:1 mixture of DG and LG with 4-PyB, no CD signals are observed (Figure S6). Cryo-TEM and SEM seem to reveal smooth fiber bundles without apparent helical sensing (Figure S7). This further emphasizes that gel chirality and fiber handedness arise from guanosine’s intrinsic chirality, uninfluenced by 4-PyB or external conditions.

The proposed assembly mechanism of this highly tunable, pH-responsive supramolecular system is depicted in Figure 2. Guanosine derivatives are known to form G-quartets via K^+^-templated assembly and through π–π stacking into highly ordered G-quadruplexes.^39,40,44^ In the present system, replacing K^+^ with Li^+^, which has a smaller ionic radius, inhibits gelation, highlighting the essential role of K^+^ ions in the gelation process. Molecular dynamics (MD) simulations support the formation of *P*-helical G-quadruplexes at high pH (Figure S8 and Video S1). The diameter of the simulated G-quadruplex nanofiber (∼2.5 nm) closely matches the diameter observed in cryo-TEM (∼3.5 nm), indicating that each G-quadruplex corresponds to a single nanofiber. This relatively simple structural organization facilitates successful MD simulations, consistent with the experimental data (Figure 1). In contrast, MD simulations at low and intermediate pH could not reproduce the multifiber and fiber-bundle morphologies observed experimentally, suggesting that these systems involve more complex secondary interactions critical for maintaining structural integrity but are beyond the scope of MD modeling.

**Figure 2:**
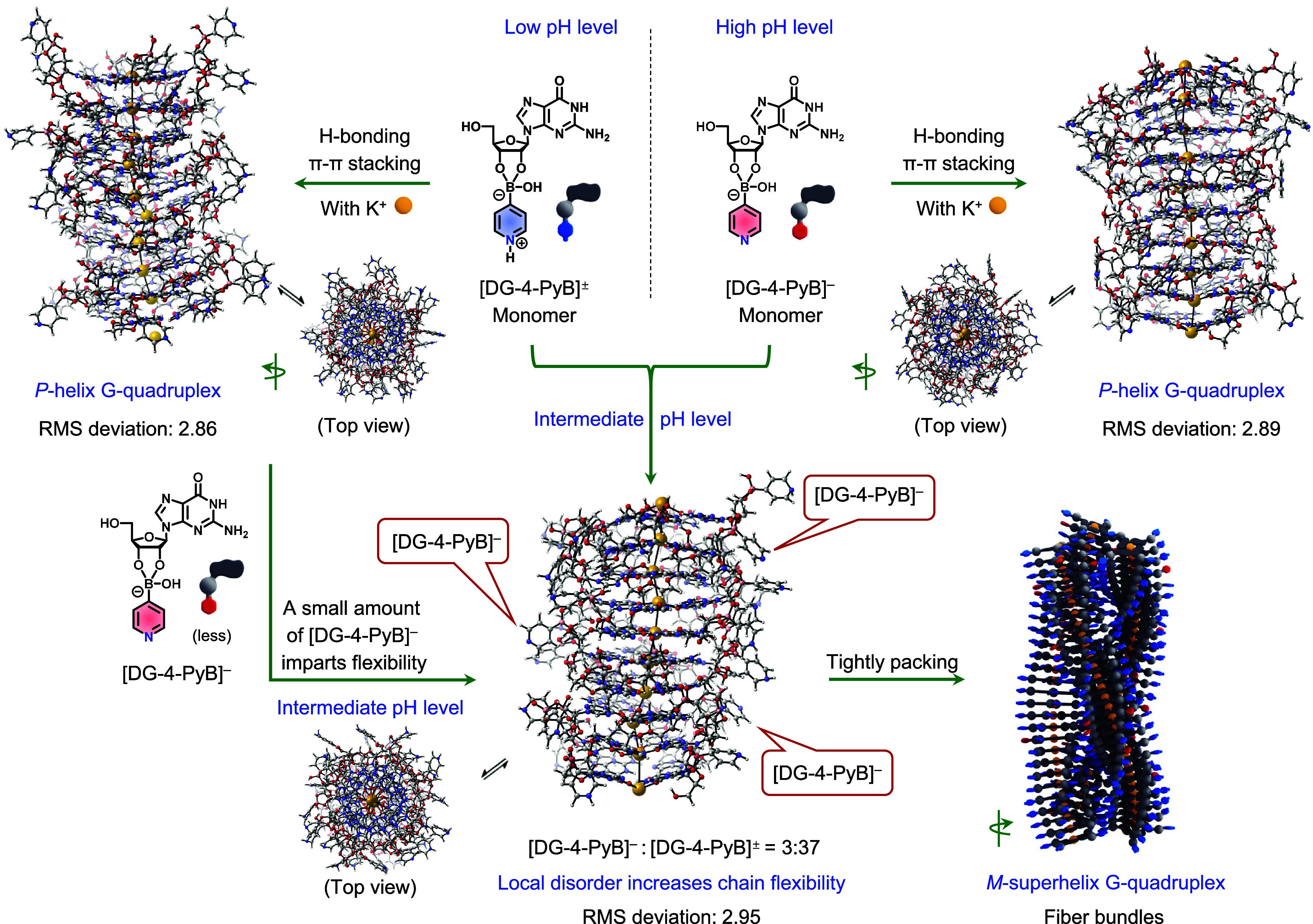
Assembly mechanisms of supramolecular gels. Top left: At low pH, the zwitterionic [DG-4-PyB]^±^ forms a *P*-helical G-quadruplex with an RMSD of 2.86. Top right: At high pH, anionic [DG-4-PyB]^−^ also assembles into a *P*-helical G-quadruplex with a similar RMSD of 2.89. Bottom: At intermediate pH, the coassembly of [DG-4-PyB]^±^ and [DG-4-PyB]^−^ produces an *M*-helical superstructure. A small amount of [DG-4-PyB]^−^ increases the structural flexibility of the G-quadruplex nanofibers (RMSD = 2.95), creating sufficient space for the tight packing of multiple single fibers. Atoms are colored as follows: red - oxygen, blue - nitrogen, gray - carbon, pink - boron, white - hydrogen, and yellow - potassium.

As a result, xTB calculations were performed on a 10-layer G-quartet model to analyze local structural changes and the root-mean-square deviation (RMSD) between the initial and optimized structures at different pH levels (Figure 2). The results show that the RMSD of the G-quadruplexes formed by anionic [DG-4-PyB]^−^ at high pH was calculated to be 2.89, similar to the RMSD of the G-quadruplexes formed by zwitterionic [DG-4-PyB]^±^ at low pH, reflecting a similar local stability. At intermediate pH, a small amount of [DG-4-PyB]^−^ induces localized disorder in [DG-4-PyB]^±^ -based G-quadruplexes, leading to hybrid structures with a higher RMSD of 2.95. These structural variations may enhance the flexibility and facilitate the tight packing of multiple fibers into *M*-superhelical bundles. This packing mechanism bears some resemblance to the assembly of collagen triple helices, where substitutions of glycine at every third residue enhance structural flexibility, enabling the formation of tightly packed, stable right-handed triple helices (Figure S9).^3^

### Self-Regulating Gel-to-Gel Transformation with Reversible Helical Switching

The unique pH-responsive behavior and inverse chiroptical signals of DG/4-PyB hydrogels inspired the development of an autonomous regulatory system capable of dynamically controlling helical structures, mimicking the adaptive regulation in biological systems.^4,45,46^ This approach was initially realized through a biocatalytic reaction network integrating urease and esterase (Figure 3). Within this system, a feedback loop was established: the pH first increased due to urease-catalyzed urea decomposition, followed by a gradual decrease from esterase-catalyzed hydrolysis of ethyl acetate (Figure 3a).^47^ The dual-stage pH modulation enabled reversible transformations of boronate esters (Figure S10) and autonomous gel-to-gel transformations with dynamic chiroptical states (Figure 3b). In a typical experiment, urea (15 μL, 2.4 M stock solution), urease (8 μL, 0.3125 g mL^–1^ stock solution), ethyl acetate (15 μL, anhydrous), esterase (10 μL, 7.5 g mL^–1^ stock solution), and KCl (3.5 μL, 1 M stock solution) were added into a 500 μL DG/4-PyB (1:1, 0.8% w/v in G, initial pH 5.8 ± 0.1) stock solution. In the latter, the zwitterionic [DG-4-PyB]^±^ species predominated, and gelation was inhibited due to the absence of K^+^. Upon the addition of catalysts and KCl, the urease activity increased the pH to 8.7 ± 0.1 within 50 min, converting [DG-4-PyB] ^±^ to the anionic [DG-4-PyB]^−^ (Figures 3c and S10). During this stage, the solution rapidly transformed into an opaque gel (within 1 min) composed of left-handed fiber bundles, as visualized by cryo-TEM and SEM (Figure 3d,e). CD spectra exhibited a negative signal, in agreement with an *M*-superhelix structure (Figure 3f,g). Rheological studies on the obtained hydrogel showed a typical viscoelastic response behavior (*G*′ > *G*″) and a significant increase in the storage modulus (*G*′) to ∼ 2200 Pa, consistent with gel densification (Figure 3h). As the pH rose to 8.7, the opaque gel fully transformed into a clear gel with positive CD signals, indicative of the *P*-helical fiber structure (Figure 3c,d,f,g). Rheological measurements revealed a reduction in storage modulus (*G*′) to ∼1000 Pa as the pH reached its maximum (Figure 3h), consistent with a network reorganization into single fibers (∼4.7 nm diameter; Figure 3e). These results demonstrated an autonomous gel-to-gel transformation with simultaneous *M*-superhelix to *P*-single helix reversal during the pH increasing process. As the gel evolves, some reservations have to be made about the relationship between the optical anisotropy and the opposite helicity of the superhelix compared to the single fiber. Selective scattering of one of the circular polarizations of light can influence the CD spectrum, especially at higher wavelengths where the molecules do not absorb light. In the rest of the paper, we assume that an inversion of helicity between an *M*-superhelix and a *P*-single helix is in line with all the experimental results. Over time, as the urea and/or urease were gradually used up and the esterase hydrolyzed the ethyl acetate, the pH began to gradually decrease, reaching to 6.0 ± 0.1 after approximately 2000 min (Figure 3c). This led to the conversion of anionic [DG-4-PyB]^−^ back to [DG-4-PyB]^±^ (Figure S10). During this stage, partial reformation of the *M*-superhelix was observed, as reflected by diminished positive CD signals and re-emergence of the aggregated fiber bundles (Figure 3e–g). Macroscopically, the gel became slightly translucent and the G′ increased to ∼6100 Pa (Figure 3d,h). Notably, crowding effects from the enzyme macromolecules likely impeded complete reassembly.^48^ By the end of the process, the hydrogel stabilized as a fully clear gel, and CD spectra converged to zero (Figure 3d,f,g). Cryo-TEM revealed single fibers again, and G′ decreased and stabilized at ∼1850 Pa (Figure 3e,h). As a result, this system demonstrated the dynamic interplay between pH modulation and assembly, achieving reversible transitions between four distinct gel states and three distinct chiroptical states: the *M*-superhelix, *P*-helix, and a macroscopic achiral state with CD = 0 (Figure 3b). The gel’s transitions from opaque to transparent to translucent to fully transparent align with transmittance changes observed via UV–vis measurements (Figure S11).

**Figure 3:**
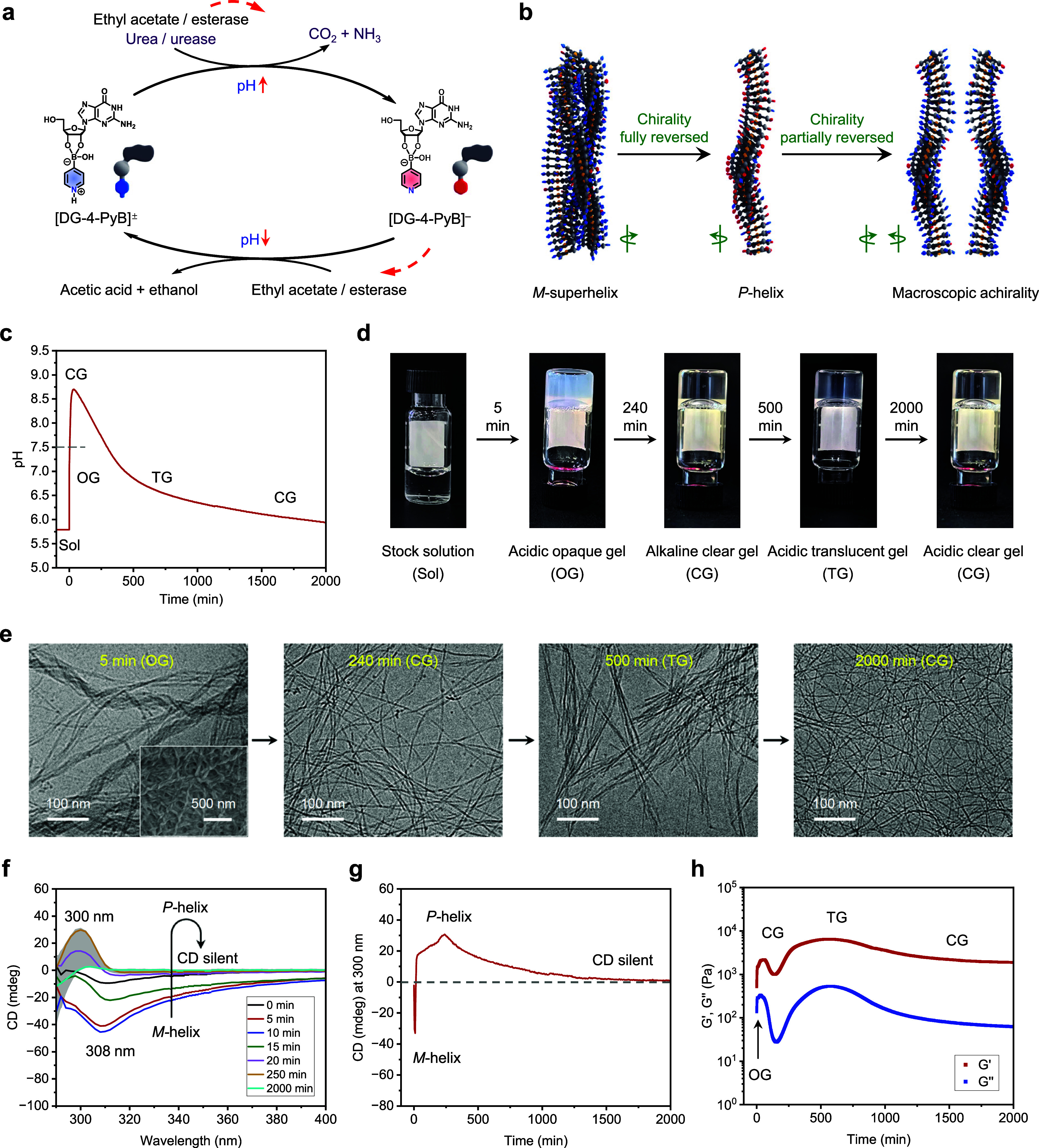
Self-regulation of the gel-to-gel-to-gel-to-gel transformation with three-state chiroptical switching. (a) Schematic illustrating the programming of a pH-sensitive boronate ester switch via a biocatalytic reaction network (BRN). The BRN, composed of urea/urease and ethyl acetate/esterase, establishes a pH feedback loop. (b) The boronate ester interacts with the BRN, driving the assembly of structural transitions. (c) Simultaneous injection of all reagents induces a rapid pH increase, followed by a delayed pH decrease due to the slow hydrolysis of ethyl acetate by esterase. (d) The system undergoes a sequential gel-to-gel-to-gel-to-gel transformation as the pH evolves. (e) Morphological changes occurred during the transformation process. Inset: SEM image of the opaque gel, displaying left-handed fibers. (f) Time-dependent CD spectra recorded during the pH evolution process. (g) CD signal intensity at 300 nm as a function of time. (h) Time-dependent rheological moduli profiles showing changes in the gel’s mechanical properties during pH evolution. Conditions: 0.8% w/v DG/4-PyB (1:1) stock solution (500 μL, initial pH 5.8 ± 0.1) mixed with urea (15 μL, 2.4 M stock solution), urease (8 μL, 0.3125 g mL^–1^ stock solution), ethyl acetate (15 μL, anhydrous), esterase (10 μL, 7.5 g mL^–1^ stock solution), and KCl (3.5 μL, 1 M stock solution).

To investigate the role of chirality, a control experiment was conducted using LG instead of DG. Under identical conditions, the LG/4-PyB system exhibited the same phase behavior as the DG/4-PyB mixture, including transformations from an opaque to a clear gel to an intermediate translucent state, and finally to a clear gel. And as expected, the CD signals exhibited opposite changes during the pH evolution process (Figure S12). Specifically, a transition from a *P*-superhelix to an *M*-helix occurred during pH elevation, and partial reformation of the *P*-superhelix was observed during pH reduction. This result underscores the crucial role of molecular chirality in determining the supramolecular assembly and chiroptical properties of the hydrogels. Crucially, these gel-to-gel transformations and reversal of optical activity occur autonomously upon chemical injection of the biocatalysts and KCl, bypassing the sol phase. This marks an innovative strategy for creating self-regulating and robust material systems with dynamic and programmable chiroptical properties.^32^

To isolate the effects of a unidirectional pH increase, a simplified system using urease and urea was studied (Figure 4).^49^ Here, DG/4-PyB (1:1, 0.8% w/v in G, 500 μL, initial pH 5.8 ± 0.1) was mixed with urea (15 μL, 2.4 M stock solution), urease (8 μL, 0.3125 g mL^–1^ stock solution), and KCl (3.5 μL, 1 M stock solution). The enzymatic decomposition of urea elevated the pH from approximately 5.8 to 8.9 over ∼90 min, driving the complete transition from an *M*-superhelix to a *P*-helix (Figure 4a–c). After the addition of urea/urease, the stock solution rapidly transformed into an opaque gel with a negative CD signal, and left-handed helical fiber bundles were revealed by cryo-TEM and SEM (Figure 4d–g). As the pH rose above 8.0, the hydrogel became transparent. A completely clear gel composed of single fibers (∼4.6 nm diameter) was observed at a final pH of 8.9 (Figure 4c,d). CD spectra showed a positive signal corresponding to a *P*-helix, while *G*′ decreased significantly from ∼2200 to ∼180 Pa, indicating a loosening of the gel network (Figure 4f–h). Video S2 captured the gel’s transition from an opaque to a clear state as the pH increased, consistent with the transmittance changes (Figure S13). This visual evidence highlights the structural stability of the gel, capable of supporting its own weight during inversion tests, emphasizing the mechanical robustness of the system, even under dynamic assembly conditions. In the LG/4-PyB control, in the presence of urea, urease, and KCl, increasing the pH also induced a transition from an opaque gel to a clear gel. However, the CD signals revealed a shift from a *P*-helix to an *M*-helix (Figure S14), reversing the chirality observed in the DG/4-PyB system. This further confirms the critical influence of molecular chirality on the assembly process.

**Figure 4:**
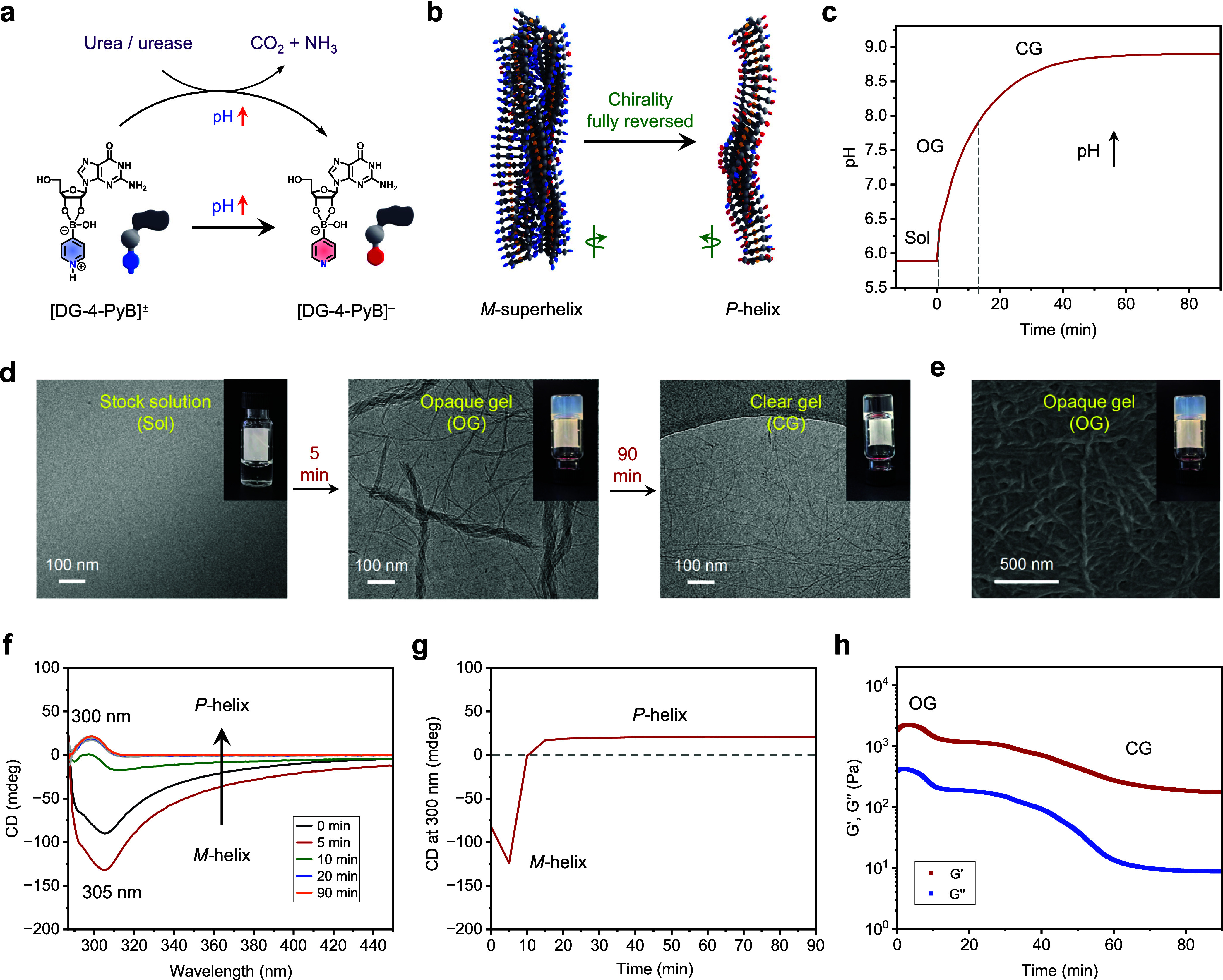
Self-regulating gel-to-gel transformation with *M*-superhelix to *P*-single helix inversion. (a) Schematic illustrating the programming of the pH-sensitive boronate ester switch through the enzymatic urea/urease system, which drives unidirectional pH elevation. (b) The boronate ester interacts with the enzymatic reaction, triggering structural transitions in the assembled gel. (c) Simultaneous injection of all of the reagents induces a rapid pH increase. (d) Morphological evolution and corresponding phase behavior (inset) during pH progression. (e) SEM image of the transient opaque gel revealing left-handed helical fibers. (f) Time-dependent CD spectra recorded during the pH evolution process. (g) CD signal intensity at 300 nm as a function of time. (h) Time-dependent rheological moduli profiles during pH evolution. Conditions: 0.8% w/v DG/4-PyB (1:1) stock solution (500 μL, initial pH 5.8 ± 0.1) mixed with urea (15 μL, 2.4 M stock solution), urease (8 μL, 0.3125 g mL^–1^ stock solution), and KCl (3.5 μL, 1 M stock solution).

To further explore the assembly dynamics under a unidirectional pH decrease, we employed the autonomous hydrolysis of β-butyrolactone, which gradually lowered the pH from alkaline to acidic conditions (Figure 5).^50^ In a typical experiment, 10 μL of β-butyrolactone was added to a 500 μL DG/4-PyB stock solution (1:1, 1.0% w/v in G) with an initial pH of 10.4 ± 0.1. As β-butyrolactone hydrolyzed, the pH of the system decreased from 10.4 to ∼4.7 over 900 min, driving the complete transition from a *P*-helix to an *M*-superhelix (Figure 5a–c). Initially, as the pH decreased to approximately 9.0, the system transitioned from a solution to a clear gel, maintaining this phase for around 200 min (Figure 5c,d). The hydrogel, composed of single fibers with a diameter of ∼4.7 nm as visualized by cryo-TEM, exhibited a positive CD signal, characteristic of a *P*-helix structure (Figure 5d–f). Rheological studies revealed that the system initially formed a solution (*G*′ < *G*″), which gradually transitioned into a gel phase (*G*′ > *G*″), consistent with macroscopic observations (Figure 5g). As the pH dropped below 6.5, the gel became opaque, accompanied by the emergence of negative CD signals indicative of an *M*-superhelical structure (Figure 5c–f). During this transition, rheological analysis showed a significant increase in the level of *G*′ to ∼2100 Pa, reflecting the formation of fiber bundles, consistent with cryo-TEM observations (Figure 5d,g). Further pH reduction to ∼5.5 caused the hydrogel to disassemble into a viscous liquid with diminished chirality and a notable decrease in *G*′ to ∼15 Pa (Figure 5c–g). Notably, the transformation from gel to viscous liquid observed through rheology lagged behind the changes detected via CD signals and visual observation. This delay may be attributed to the time required for the bulk mechanical properties to reflect microstructural changes, highlighting the distinct time scales of these processes. Despite this lag, the overall phase change sequence (solution → clear gel → opaque gel → viscous liquid) remained consistent across analytical methods (Figure S15). In the LG/4-PyB control, the pH decreases induced the same phase behavior as that of the DG/4-PyB mixture. However, the CD signals exhibited completely opposite changes during the pH evolution process (Figure S16). The highly acidic conditions led to the disassembly of the final gels.

**Figure 5:**
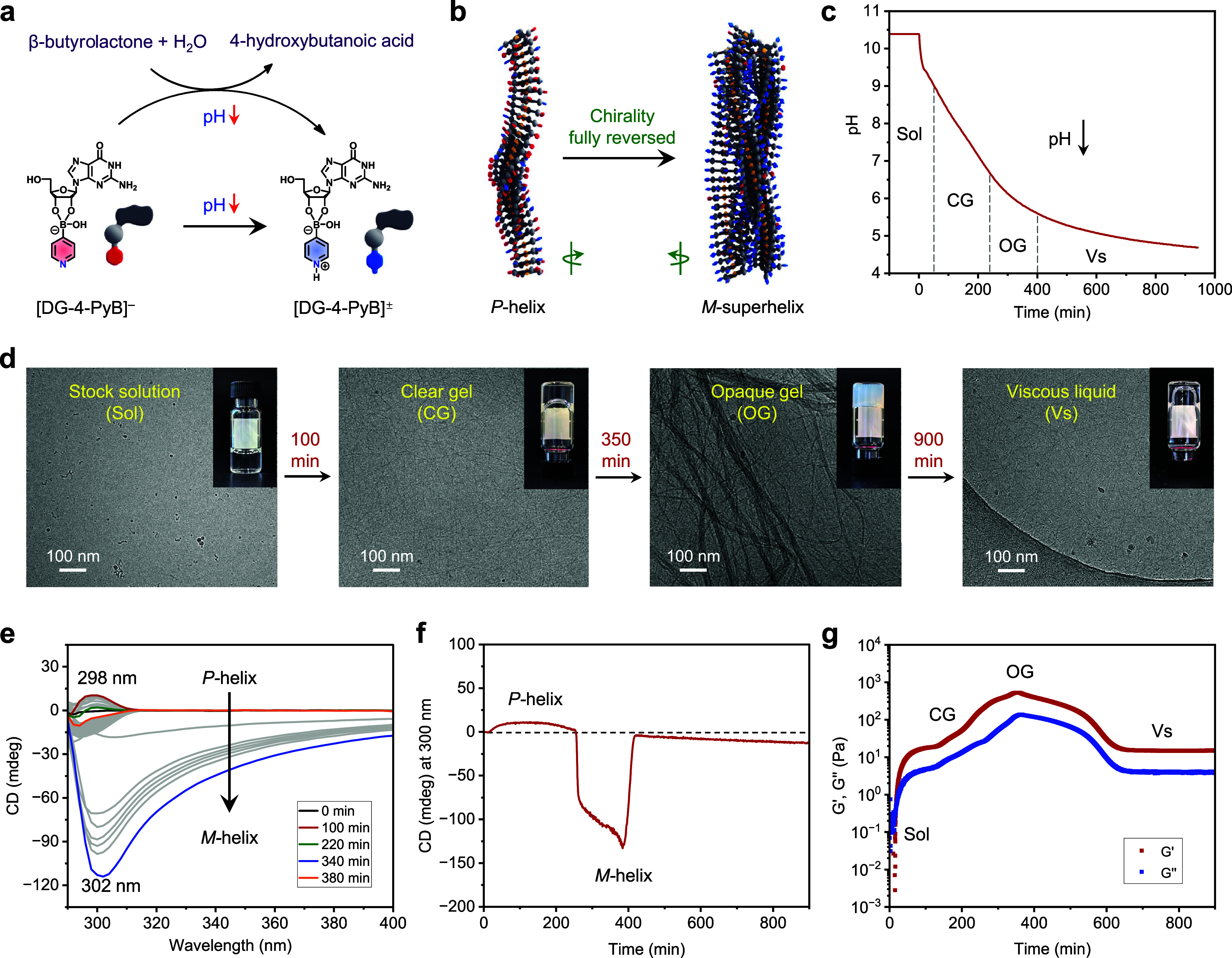
Self-regulating gel-to-gel transformation with *P*-helix to *M*-helix inversion. (a) Schematic illustrating the programming of the pH-sensitive boronate ester switch via the hydrolysis of β-butyrolactone, driving a unidirectional pH decrease. (b) The boronate ester interacts with the hydrolysis reaction, triggering structural transitions in the assembled gel. (c) Simultaneous injection of all of the reagents induces a rapid pH decrease. (d) Morphological evolution and corresponding phase behavior (inset) during the pH progression. (e) Time-dependent CD spectra were recorded during the pH evolution process. (f) CD signal intensity at 300 nm as a function of time. (g) Time-dependent rheological moduli profiles during pH evolution. Conditions: 1.0% w/v DG/4-PyB (1:1) stock solution (500 μL, initial pH 10.4 ± 0.1) mixed with β-butyrolactone (10 μL).

The three systems collectively highlight the versatility and complexity of gel-to-gel transformations, driven by pH modulation. The dynamic feedback system achieves bidirectional control of chirality but exhibits partial reversibility during pH reduction, likely constrained by steric hindrance from enzymatic macromolecules.^48^ In contrast, the monotonic pH increase system achieves a complete *M*-superhelix to *P*-helix transition, while the monotonic pH decrease system accomplishes a full *P*-helix to *M*-superhelix reversal, highlighting complementary and unrestricted assembly dynamics. The pH-dependent transition between single fibers and bundled superhelices is closely related to the charge distribution of the boronic ester. At low pH, the pyridine group is protonated while the boronate remains anionic, forming a zwitterionic structure ([DG-4-PyB]^±^) with a high dipole moment but neutral net charge. This significantly enhances dipole–dipole interactions and reduces electrostatic repulsion between fibers. This promotes interfiber association via π–π stacking, hydrogen bonding, and possibly dipole–dipole alignment, leading to the formation of bundled superhelical structures. In contrast, at high pH, the deprotonated pyridine–boronate ester ([DG-4-PyB]^−^) bears a net negative charge, increasing fiber–fiber electrostatic repulsion and favoring the formation of well-dispersed single fibers. During the structure transition process, a cooperative, all-at-once inversion of helices is unlikely due to structural constraints within the gel matrix. Instead, we propose that the inversion proceeds gradually and asynchronously, involving localized disruptions of the supramolecular order. Transient intermediates, such as disordered, partially relaxed helices, oligomers, or monomers, may form as individual helices undergo stepwise uncoiling or misalignment before reorganizing into the opposite handedness. These species likely remain embedded within the network and may not accumulate to detectable levels due to the slow kinetics and overlapping time scales of helix reformation and network relaxation.

## Conclusions

In this work, we developed a new supramolecular hydrogel system that autonomously undergoes gel-to-gel transformations and reversible superhelix-to-single helix inversion with biomimetic adaptability, bypassing the sol phase. Leveraging the unique interaction between 4-pyridinylboronic acid and guanosine alongside biocatalytic reaction networks for pH regulation, the system achieves dynamic control over chirality, morphology, and mechanical properties. Structural analysis revealed hierarchical assembly and inversion of optical activity between *M*-superhelices and *P*-helices, dictated by guanosine’s intrinsic chirality. The dynamic feedback loop, along with monotonic pH modulation systems, provides a comprehensive understanding of hierarchical assembly and chirality control under diverse conditions. This study establishes a novel platform for self-regulating, biomimetic chiral materials with broad applications in adaptive optics, responsive biomaterials, and beyond. The ability to mimic collagen-like hierarchical organization and dynamic adaptability positions these gels as promising candidates for advancing next-generation programmable soft materials. Future studies could explore additional regulatory mechanisms and extend the platform to other functional materials, further advancing the field of programmable soft matter.

## Supplementary Material







## Notes

NMR spectra, additional CD spectra and cryo-TEM/SEM images, experimental details, materials and methods (PDF)
Simulated P-helical single fiber formed by­[DG-4-PyB]^–^ monomers (Video S1) (MP4)
Self-regulating gel-to-gel transformation driven by urea and urease (Video S2) (MP4)
