# Supplementary material for: Self-Regulating Hydrogel with Reversible Optical Activity in Its Gel-to-Gel Transformation
Source: J Am Chem Soc. 2025 May 9;147(20):17361–71. doi: 10.1021/jacs.5c03844 (PMC12100726; doi:10.1021/jacs.5c03844)
Supplement: Supplementary file 1 [file ja5c03844_si_001.pdf]

Supporting Information

Self-Regulating Hydrogel with Reversible Optical Activity in its Gel-to-Gel Transformation

Jingjing Li,[1,2,3] Fang Yin,[2] Jianhong Wang,[2] Huachuan Du,[2] Fan Xu,[2] Stefan Meskers,[2] Yudong Li,[2] Stefan Wijker,[2] Yu Peng,[1] Riccardo Bellan,[2] Ghislaine Vantomme,[2] Jian Song,[4,*] Chun-Sen Liu,[1,*] and E. W. Meijer[2,*]

[1] *College of New Energy, Zhengzhou University of Light Industry, Zhengzhou 450002, China.*

[2] *Institute for Complex Molecular Systems and Laboratory of Macromolecular and Organic Chemistry, Eindhoven University of Technology, Eindhoven 5600 MB, Netherlands.*

[3] *School of Chemistry and Chemical Engineering, Henan University of Technology, Zhengzhou 450001, China.*

[4] *School of Chemical Engineering and Technology, Tianjin University, Tianjin 300350, China.*

** Authors to whom correspondence should be addressed.*

Email: songjian@tju.edu.cn; nkchunsenliu@163.com; e.w.meijer@tue.nl.

The PDF file includes:

Materials and Methods

Supplementary Figures S1 to S16

Supplementary Text

References

Other Supplementary Material for this manuscript includes the following:

Video S1. Simulated *P*-helical single fiber formed by $[DG\text{-}4\text{-}PyB]^{-}$ monomers.

Video S2. Self-regulating gel-to-gel transformation driven by urea and urease.

Materials and Methods

1. Materials

D-Guanosine (DG, 98%) and 4-pyridinylboronic acid (4-PyB) (98%) were purchased from TCI. L-Guanosine (LG, 98%) was purchased from BDL. KOH (99.98%), β-Butyrolactone (98%), urea (≥98%), and urease from Canavalia ensiformis (Jack bean) (Type C-3, powder, ≥600,000 units/g solid), esterase from porcine liver (lyophilized powder, ≥15 units/mg solid), was purchased from Sigma-Aldrich. Ethyl acetate (ACS reagent, ≥99.5%), was purchased at Biosolve (Valkenswaard, the Netherlands) and dried with molecular sieve before use. All other chemicals used in this study were analytical grade reagents and used as received. The water used in sample preparation was MQ water (18.2 MΩ·cm).

2. Characterization

pH Measurement: The pH of the gels was monitored using a pH meter (FiveEasy Plus FE28-micro, Mettler Toledo). Prior to measurement, the pH meter was calibrated with standard buffer solutions at pH 4.00, 7.00, and 9.21.

Nuclear Magnetic Resonance (NMR): ^{1}H NMR spectra were recorded on a Bruker 400 MHz Ultrashield spectrometer. ^{11}B NMR spectra were obtained at Bruker 500 MHz Ultrashield spectrometer. Deuterated solvents D_2O were used in each case. The background signal from the NMR probe and tube for ^{11}B NMR was eliminated through backward linear FID prediction. Chemical shifts for ^{1}H NMR were referenced to the residual solvent peak, while those for ^{11}B NMR were referenced to BF_3OEt_2.

Cryogenic transmission electron microscopy (cryo-TEM): For cryo-TEM analysis, Quantifoil grids (R 2/2, Cu, Quantifoil Jena grids, Quantifoil Micro Tools GmbH) were employed. Prior to sample application, grids underwent surface plasma treatment at 5 mA for 40 s using a Cressington 208 carbon coater. A 3 μL volume of the sample was applied to the grid, which was then placed in an automated vitrification robot (FEI Vitrobot Mark IV), operating at 22 °C and 100% relative humidity. Excess sample was removed by blotting for 3.5 s with filter paper at a blotting force of −3. The thin film was vitrified by plunging the grid into liquid ethane just above its freezing point and then transferred into liquid nitrogen. The vitrified grids were subsequently transferred to the vacuum chamber of a Glacios (Thermo Fisher), equipped with a field emission gun operating at 200 kV, a Falcon 4i direct electron detector, an autoloader station, and a post-column Gatan energy filter. Imaging of the vitrified films was performed in the Glacios at temperatures below −170 °C. Images were captured under low-dose conditions, with defocus values of −40 μm, −10 μm, and −5 μm, at a magnification of 8,500 and 24,000.

ESI Q-TOF MS: High-resolution electrospray ionization mass spectrometry (ESI-MS) was performed on a Q-Tof Ultima Global mass spectrometer (Micromass, Manchester, UK) with a Z-spray source. Electrospray ionization was conducted in positive mode with a needle voltage of 3 kV. A 0.1 mg

mL^{-1} aqueous solution was directly and continuously infused at a flow rate of 5 μL min^{-1} using a syringe pump, with the solution maintained at room temperature. The source block temperature was set to 60 °C, and the desolvation gas was heated to 80 °C. Argon was used as the collision gas, and the cone voltage was fixed at 35 V. The mass spectrometer operated within the mass range of 100−1200 amu. No acid was added during these experiments to induce the formation of charged species, leading to the detection of primarily complexed species.

Rheology: Rheological measurements were performed using a TA Instruments DHR-3 rheometer with a parallel plate geometry (20 mm diameter, 1.0 mm gap). Hydrogel precursor hot solution was dropped onto the plate, and the sample was allowed to equilibrate for 30 minutes at 25 °C before testing. The temperature was controlled using a Peltier system, with an oil trap to prevent evaporation. The storage modulus (G′) and loss modulus (G″) were measured over a strain range of 0.1% to 1000% at 1 Hz for the strain sweep, and 1% strain at a frequency range of 1 to 100 rad s^{-1} for the frequency sweep.

Circular Dichroism (CD) and UV-vis Spectroscopy: Circular dichroism (CD) and UV-vis absorption spectroscopy were conducted using a JASCO J-815 CD spectrometer. To ensure repeatability and minimize cooling rate effects, single measurements for the pH 10.0 and pH 7.5 supramolecular gels were taken at 25 °C after heating the samples to 70 °C and cooling them to 25 °C at a controlled rate of 0.5 °C min^{-1}. For the pH 5.0 supramolecular gels, single measurements were performed at 32 °C to avoid linear dichroism (LD) effects, following a similar heating/cooling process. For self-regulating gels, time-variable CD measurements were carried out at 25 °C. UV-vis measurements were performed concurrently with all CD tests. A path length of 0.5 mm was used for all measurements.

Scanning Electron Microscopy (SEM): SEM images were captured using a FEI Quanta 200 3D FEG SEM at 5.00 kV voltage (Thermo Fisher Scientific, USA). Hydrogel samples were freeze-dried prior to analysis. Xerogel samples were gold-coated before SEM imaging to enhance conductivity.

3. Preparation of the supramolecular hydrogels

The supramolecular hydrogels were prepared by mixing specific amounts of DG (or LG), 4-PyB, and a suitable aqueous solution in a clean vial. The mixture was heated to 100 °C for 2 minutes to completely dissolve the components, and subsequently allowed to cool at room temperature in ambient air to facilitate the formation of a hydrogel.

DG/4-PyB Hydrogel at pH 10.0 (1.5% w/v): DG (15.0 mg, 0.053 mmol), 4-PyB (6.5 mg, 0.053 mmol), and KOH solution (pH = 13.0, 1.0 mL) were used. The resulting hydrogel was fully transparent, with a final pH of ~10.0.

DG/4-PyB Hydrogel at pH 7.5 (0.6% w/v): DG (12.0 mg, 0.042 mmol), 4-PyB (5.2 mg, 0.042 mmol), and KOH solution (pH = 12.0, 2.0 mL) were used. The resulting hydrogel was opaque, with a final pH of ~7.5.

DG/4-PyB Hydrogel at pH 5.0 (1.5% w/v): DG (15.0 mg, 0.053 mmol), 4-PyB (6.5 mg, 0.053 mmol), Milli-Q water (1.0 mL), and KCl solution (26.5 μL, 1 M stock) were used. The molar ratio of DG:4-PyB:K^+ was maintained at 1:1:0.5. The resulting hydrogel was clear, with a final pH of ~5.0.

Other hydrogels were prepared similarly. The pH of the aqueous solution was adjusted based on the concentrations of D-guanosine (DG) and 4-PyB to ensure consistency in the final gel pH. For LG/4-PyB hydrogels, DG was replaced with LG, while all other conditions remained unchanged.

4. Preparation of the self-regulating supramolecular hydrogels

The self-regulating supramolecular hydrogels were prepared using a pH feedback system as a representative example. A 500 μL stock solution of 0.8% w/v DG/4-PyB (1:1) mixture in Milli-Q water was used. To this solution, anhydrous ethyl acetate (15 μL), urea (15 μL, 2.4 M stock solution), esterase (10 μL, 7.5 $g \cdot mL^{-1}$ stock solution), urease (8 μL, 0.3125 $g \cdot mL^{-1}$ stock solution), and KCl (3.5 μL, 1 M stock solution) were added and mixed using vortex agitation for 30 seconds.

This process resulted in a rapid transformation from a solution to an opaque hydrogel, which progressively transitioned to a fully transparent gel, then to a translucent gel, and finally back to a fully transparent gel over time. The entire cycle was monitored using a pH meter (FiveEasy Plus FE28, Mettler Toledo), CD, and rheological measurements.

The preparation of other self-regulating gels driven by urea/urease or β-butyrolactone followed a similar procedure. For LG/4-PyB gels, DG was replaced with LG, while all other conditions remained unchanged.

5. pK_a calculation

The pK_a was calculated using the following thermodynamic cycle:[1,2]

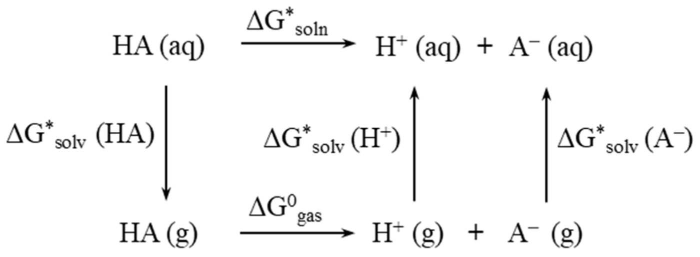

Scheme S1. Thermodynamic cycle used in the calculation of pK_a of [DG-4-PyB]$^-$.

All initial geometries for density functional theory (DFT) calculations were generated using GaussView software. The optimization of these geometries was carried out with Gaussian 16 software at the B3LYP/6-311G (d,p) level with empirical dispersion corrections (GD3BJ).[3-5] Vibrational analysis was also performed at the same level to ensure the absence of imaginary frequencies in all optimized geometries.

The pK_a values were calculated using the following formula:[1]

$$\mathrm{p}K_\mathrm{a} = \frac{G_{gas}(A^-) - G_{gas}(HA) + \Delta G_{solv}(A^-) - \Delta G_{solv}(HA) - 269.0}{1.3644} \quad (1)$$

Here, the gas-phase free energies of HA and A^- were determined using the CBS-QB3 method, while their solvation-free energies were calculated at the M052X/6-31G* level with the SMD solvation model (scrf = SMD).[6]

6. Molecular dynamics (MD) simulations

The helical structure model of the G-quartet was constructed using custom-written scripts, incorporating 30 layers of G-quartets to form a G-quadruplex nanofiber. The single-layer structure was optimized using Gaussian 16 software with the B3LYP/6-311G(d,p) method and the empirical dispersion correction (GD3BJ). Vibrational analysis was performed at the same level to confirm the absence of imaginary frequencies in all optimized geometries.

The wavefunction of the optimized structure was subsequently analyzed using Multiwfn 3.7 software.[7] GAFF forcefield parameters for the single-layer structure were generated using the Sobtop software.[8,9] Finally, GROMACS 2022.5 was employed for further optimization and MD production simulations, conducted under an NVT ensemble for 10 ns.[10]

7. xTB simulations

Helical models of G-quartet structures were constructed using custom-written scripts, with each system containing 10 layers of G-quartets. The structures were first cleaned using GaussView and subsequently optimized with xTB 7.1.1 at the GFN2 level, employing a water implicit solvent model.[11,12] Molecular graphs were visualized using CYLView.[13]

Supplementary Figures

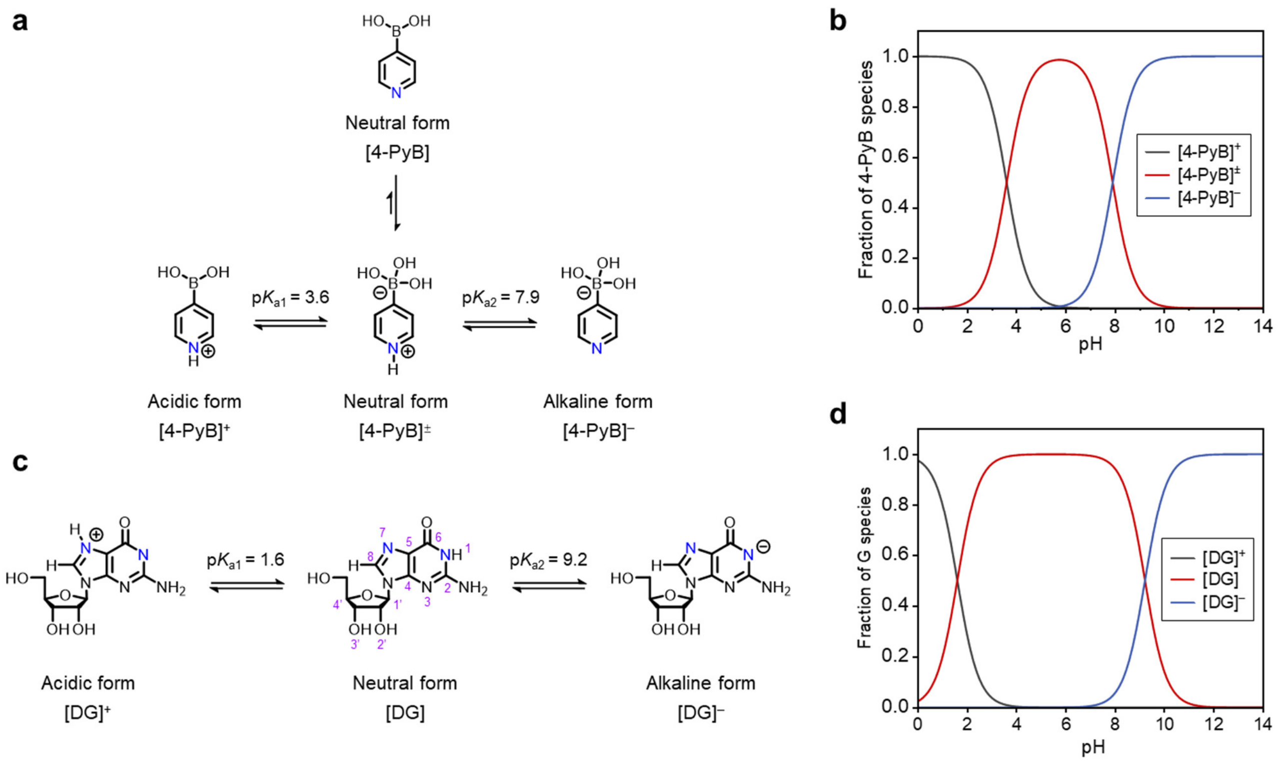

Figure S1. Chemical structures of (**a**) 4-pyridinylboronic acid (4-PyB) and (**c**) D-Guanosine (DG). Distribution curves of (**b**) 4-PyB species and (**d**) DG species. pK_a data were obtained from the references.[14-16]

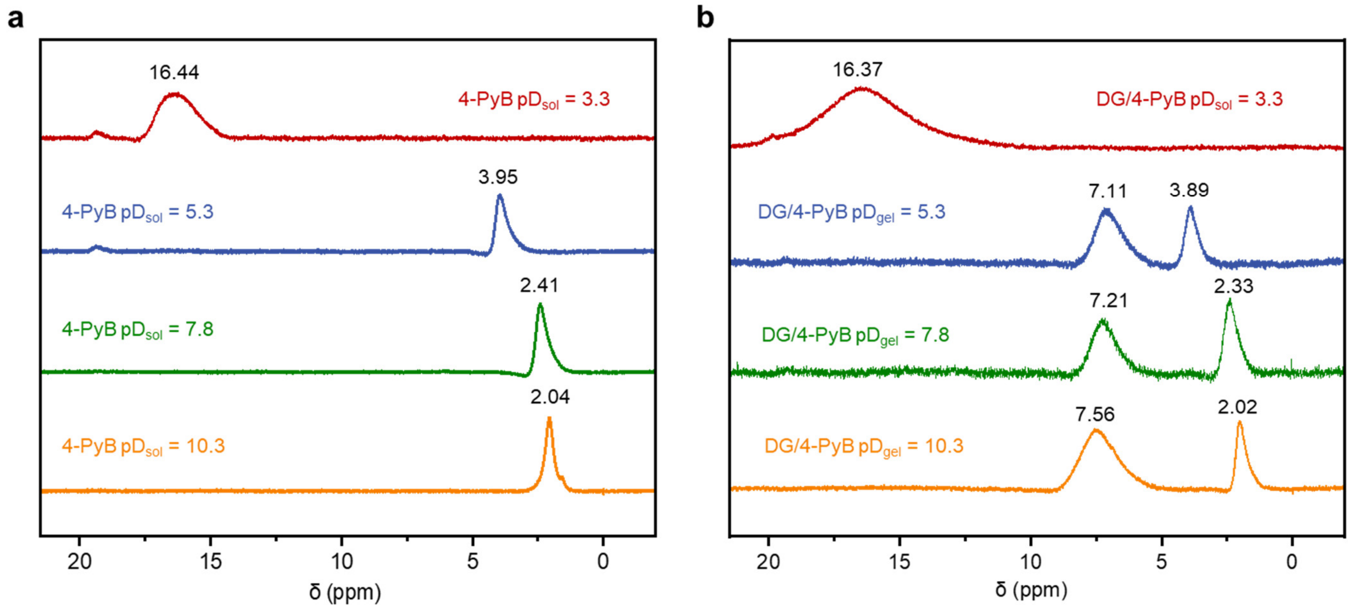

Figure S2. ^{11}B NMR spectra of 4-PyB (**a**) and its mixture with DG (**b**) under different pH conditions (pD = pH + (0.3~0.4)). Since 4-PyB was not completely soluble in water, the samples were filtered before measurement.

The ^{11}B NMR spectrum of free 4-PyB at pD 3.3 shows a low-field signal at 16.44 ppm, which shifts to a high-field signal at 2.04 ppm at pD 10.3 (Figure S2a). This indicates that boron in 4-PyB transitions from an sp^2-hybridized trigonal planar configuration at acidic pH to an sp^3-hybridized tetrahedral configuration, forming the anionic $[4\text{-PyB}]^-$ species after deprotonation by KOH.[17] Notably, at weakly acidic and neutral pH (pD 5.3 and 7.8), 4-PyB predominantly adopts an sp^3-hybridized boron configuration, consistent with previous reports.[14] When mixed with DG at pD 3.3, no boronate ester formation is observed, as only an sp^2 boron signal at 16.37 ppm is detected. When mixed with DG at pD > 5.0, a new sp^3 boron signal at 7–8 ppm appears (Figure S2b). This signal originates from the formation of boronate ester,[18,19] which facilitates gelation under suitable conditions (pD 5.3-10.3). The boronate ester formation is further confirmed by ^1H NMR and mass spectrometry (MS) analyses.

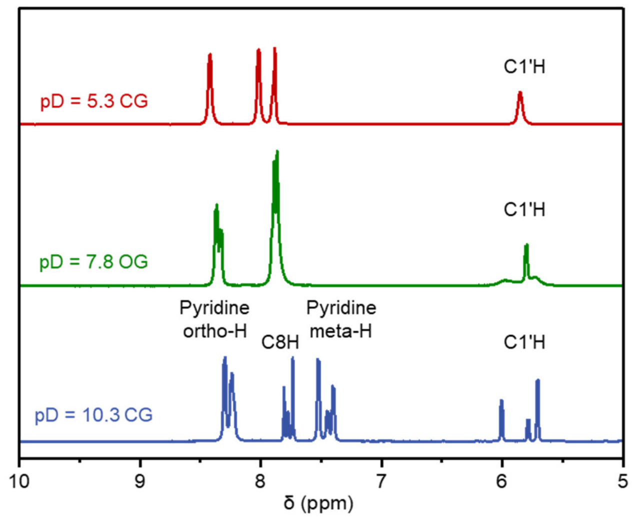

Figure S3. ^1H NMR spectra of the gels under different pH conditions.

At pD 10.3, the gel sample display three groups of sharp peaks at 5.5–6.2 ppm and 7.6–7.9 ppm, corresponding to the C1'H and C8H protons of the guanosine segments, respectively.[20] Peaks at 7.3–7.6 ppm and 8.1–8.4 ppm are assigned to the aromatic protons of the pyridine moiety.[15] The appearance of these multiple peak groups indicates the formation of boronate ester,[20] consistent with the observations in the ^{11}B NMR spectrum (Figure S2). As the pD decreases to 7.8 and further to 5.3, the peak shapes and distributions change significantly, with only one distinct group of peaks remaining at pD 5.3. Attempts to accurately assign each peak included various supplementary experiments, such as variable-temperature NMR, multidimensional NMR, and theoretical NMR predictions. However, these efforts were ultimately unsuccessful due to the system being a complex mixture with subtle differences. As a result, the changes in the ^1H NMR spectra provide only a qualitative insight into the pH-dependent evolution of chemical speciation.

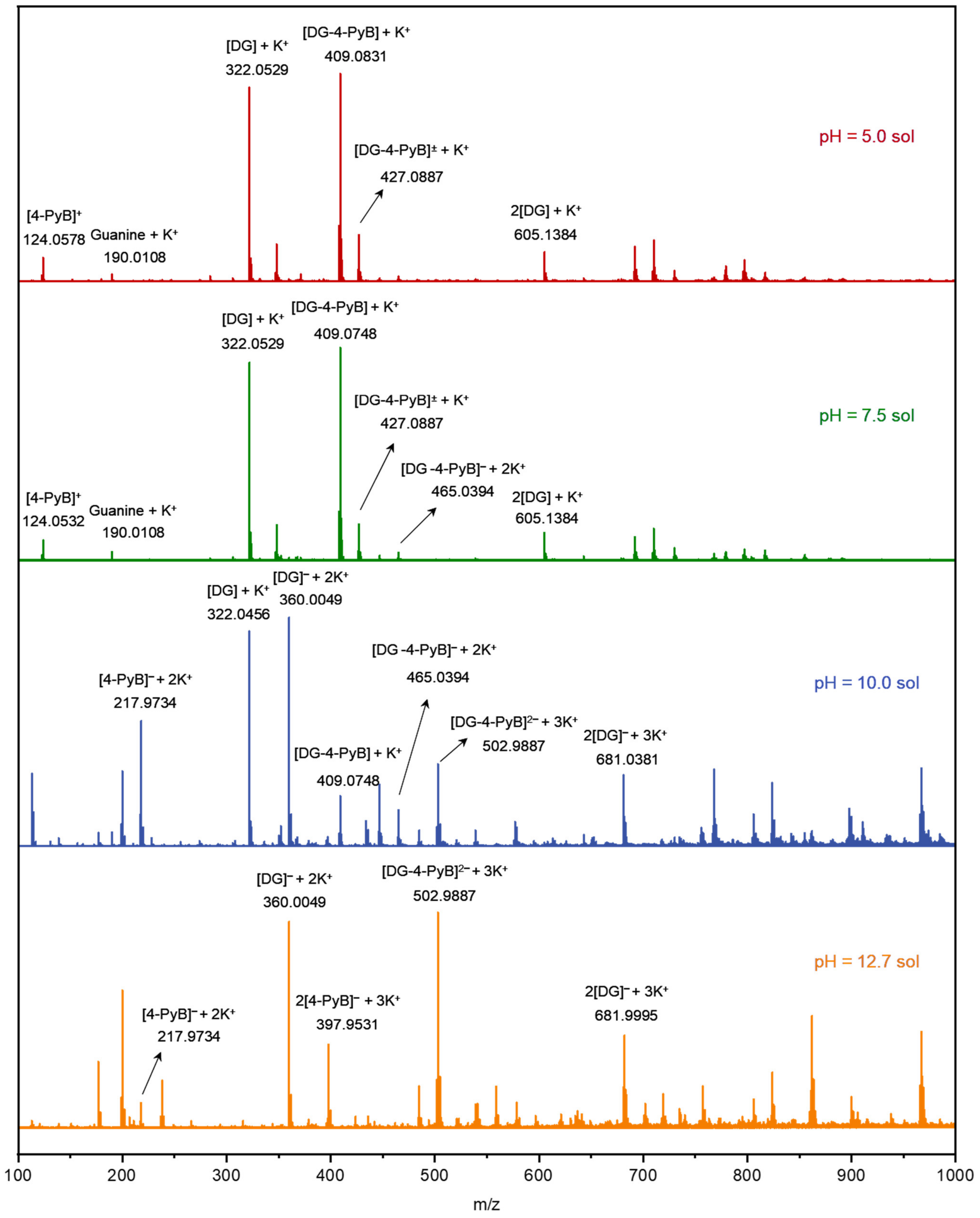

Figure S4. Mass spectra of the DG/4-PyB mixture (0.1 mg mL^{-1}) under varying pH conditions, illustrating the transformation of species from [DG] to $[DG]^{-}$, $[DG\text{-}4\text{-}PyB]^{\pm}$ to $[DG\text{-}4\text{-}PyB]^{-}$, and subsequently to $[DG\text{-}4\text{-}PyB]^{2-}$ as the pH increases.

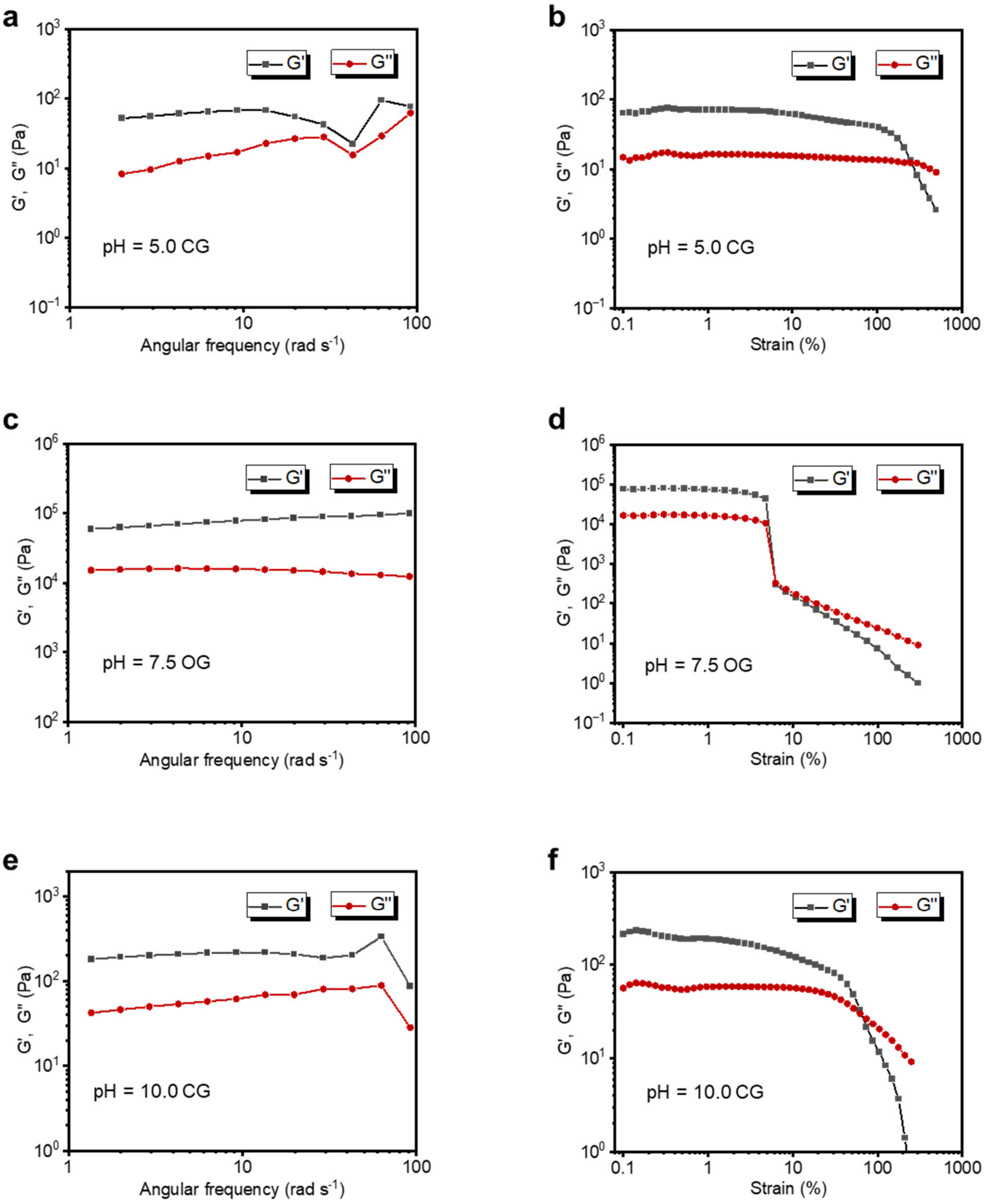

Figure S5. Rheological analysis of DG/4-PyB hydrogels at a concentration of 2% w/v and pH values of 5.0, 7.5, and 10.0: (**a**), (**c**), and (**e**) represent dynamic frequency sweeps, while (**b**), (**d**), and (**f**) correspond to dynamic strain sweeps.

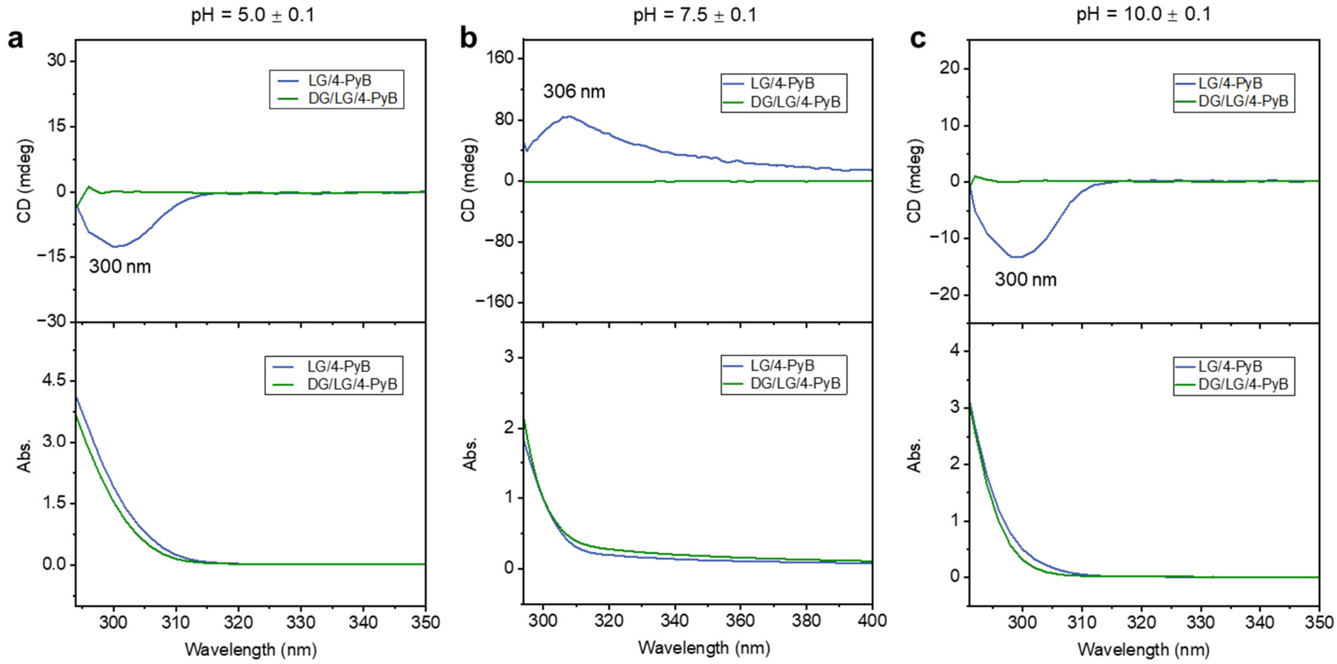

Figure S6. pH-dependent CD and absorption spectra of thermogels formed by the LG/4-PyB (1:1) mixture and the DG/LG/4-PyB (0.5:0.5:1) mixture: (**a**) Gels at pH 5.0 with a total guanosine concentration of 1.5% w/v (53 mM), (**b**) Gels at pH 7.5 with a total G concentration of 0.6% w/v (21 mM), and (**c**) Gels at pH 10.0 with a total G concentration of 1.5% w/v (53 mM).

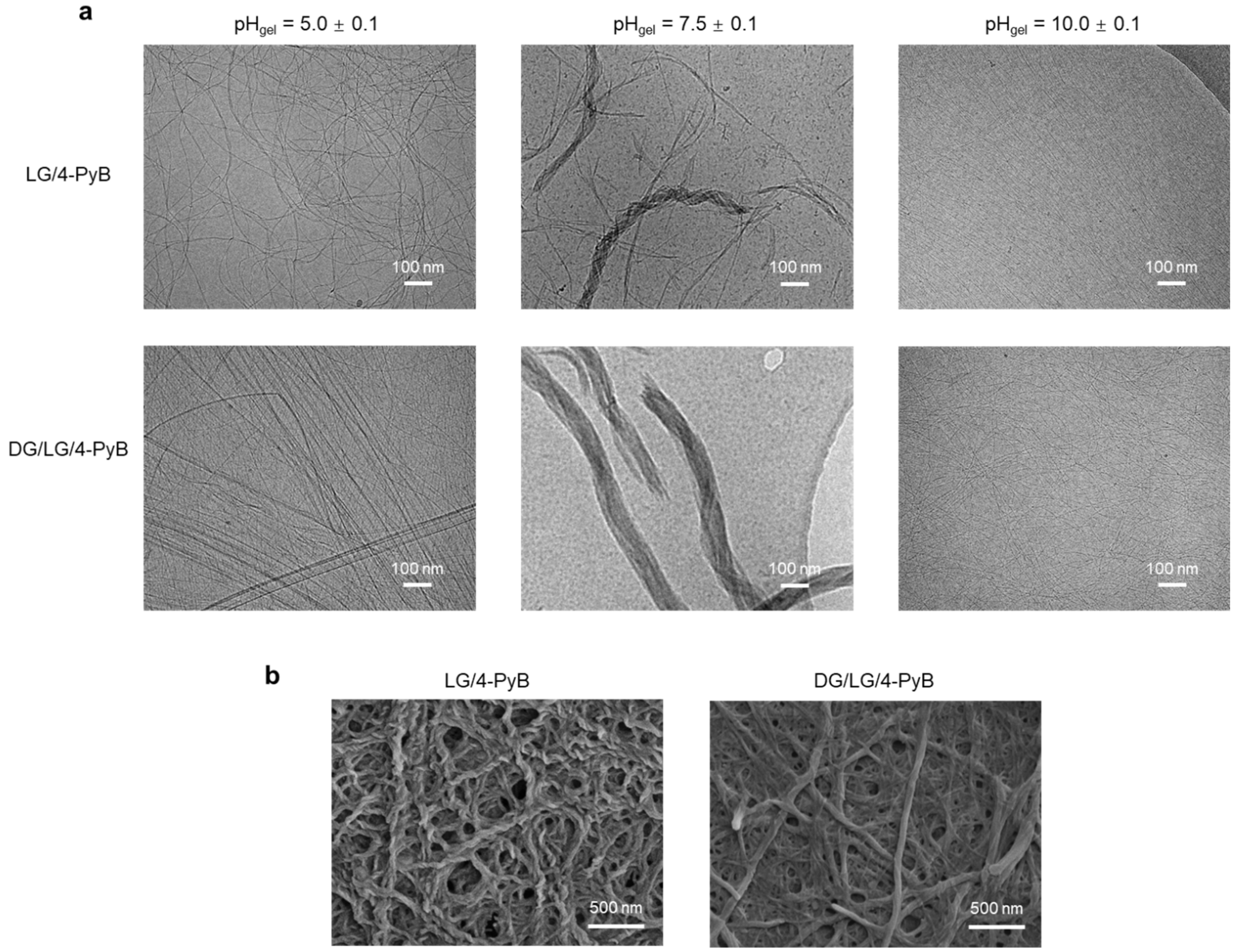

Figure S7. (**a**) pH-dependent morphological evolution of thermogels formed by the LG/4-PyB (1:1) mixture and the DG/LG/4-PyB (0.5:0.5:1) mixture, observed using Cryo-TEM. (**b**) SEM images of gels formed at pH ~7.5 by the LG/4-PyB (1:1) mixture and the DG/LG/4-PyB (0.5:0.5:1) mixture. Conditions: Gels at pH 5.0 and 10.0 were prepared with a total guanoisine concentration of 1.5% w/v (53 mM), while gels at pH 7.5 were prepared with a total guanoisine concentration of 1.0% w/v (35 mM).

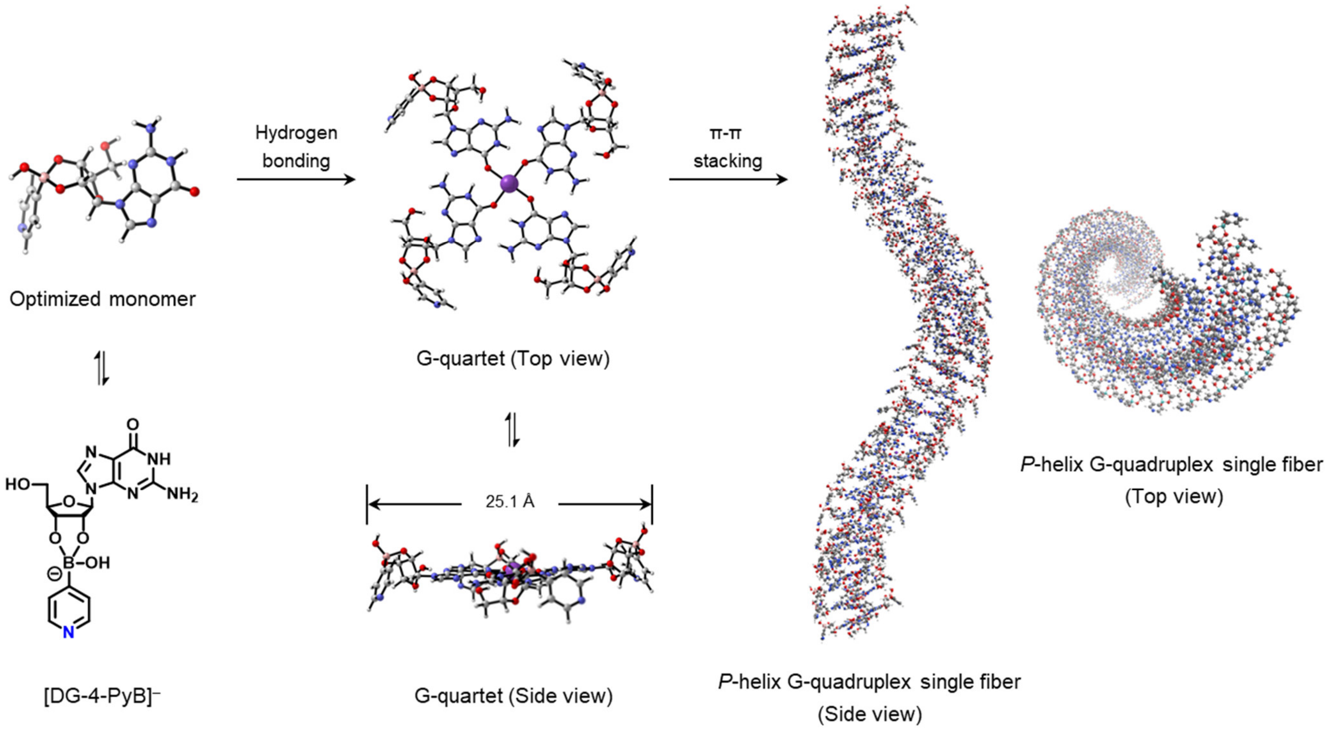

Figure S8. Simulated *P*-helical single fiber formed by [DG-4-PyB]⁻ monomers (Video S1). Atoms are colored as follows: red - oxygen, blue - nitrogen, gray - carbon, pink - boron, white - hydrogen, and purple - potassium.

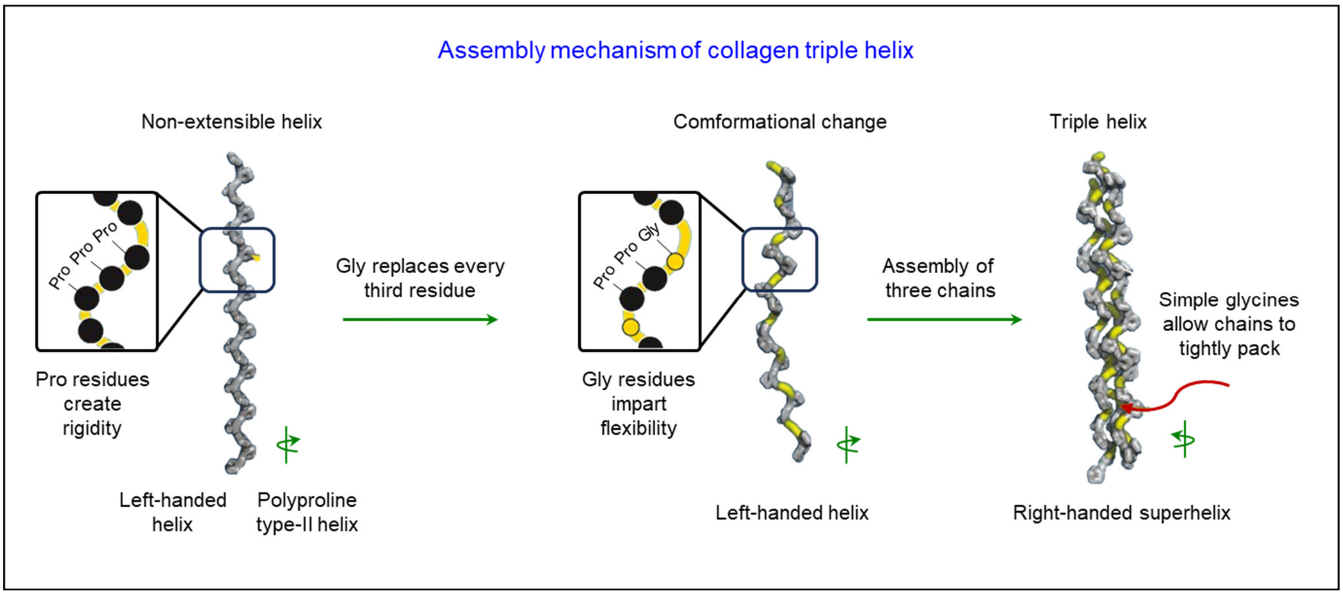

Figure S9. Assembly mechanism of the collagen triple helix. Figure adapted with permission from ref[21] Copyright 2018 Elsevier.

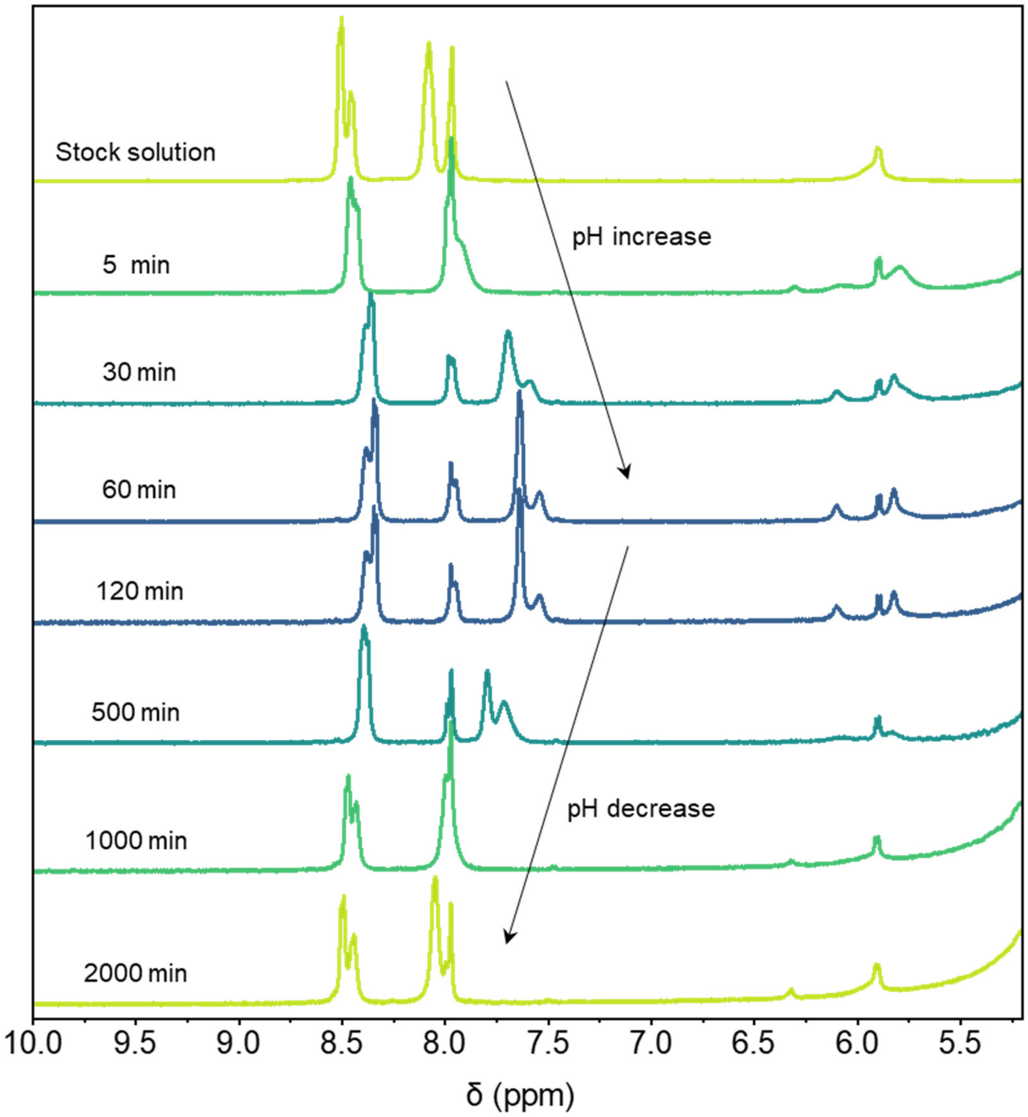

Figure S10. Evolution of the ^{1}H NMR spectra of the self-regulating gels in response to the pH feedback loop. Conditions: 0.8% w/v DG/4-PyB (1:1) stock solution (500 μL) mixed with urea (15 μL, 2.4 M stock solution), urease (8 μL, 0.3125 g mL^{-1} stock solution), ethyl acetate (15 μL, anhydrous), esterase (10 μL, 7.5 g mL^{-1} stock solution), and KCl (3.5 μL, 1 M stock solution).

As the pH first increased and then decreased, the ^{1}H NMR spectra of the self-regulating gels shifted to higher fields, with some peaks initially splitting and then returning to their original positions. This change indirectly reflects the pH-dependent chemical speciation changes. However, as discussed in Fig. S3, the clear assignment of each peak was unsuccessful, so this result only provides qualitative evidence.

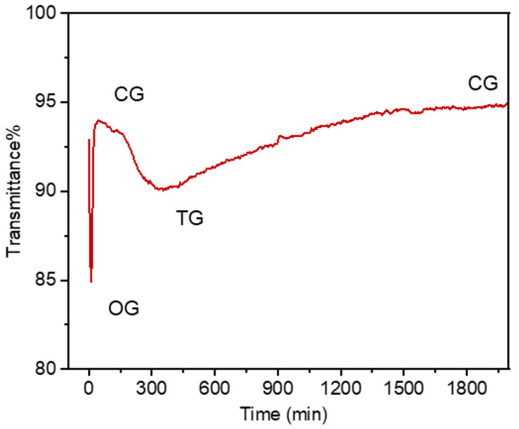

Figure S11. Time-dependent transmittance of the DG/4-PyB mixture under a pH feedback loop, measured at 400 nm with a 0.5 mm path length. Conditions: 0.8% w/v DG/4-PyB (1:1) stock solution (500 μL, initial pH 5.8 ± 0.1) mixed with urea (15 μL, 2.4 M stock solution), urease (8 μL, 0.3125 g mL^{-1} stock solution), ethyl acetate (15 μL, anhydrous), esterase (10 μL, 7.5 g mL^{-1} stock solution), and KCl (3.5 μL, 1 M stock solution).

The time-dependent transmittance profile clearly reveals the dynamic structural evolution of the gel under a pH feedback loop. Initially, the transmittance drops to approximately 85%, corresponding to the formation of an opaque gel (OG). This is followed by a rapid increase to around 95%, indicating the emergence of a clear gel (CG) phase. Subsequently, the transmittance decreases again to a minimum of ~89% as the system transitions into a translucent gel (TG) stage. Over time, the transmittance steadily rises and eventually stabilizes at around 95%, marking the formation of a uniform and fully transparent gel (CG). This progressive optical transition is consistent with the observed gel–gel transformation and underscores the time-dependent self-assembly behavior of the DG/4-PyB system.

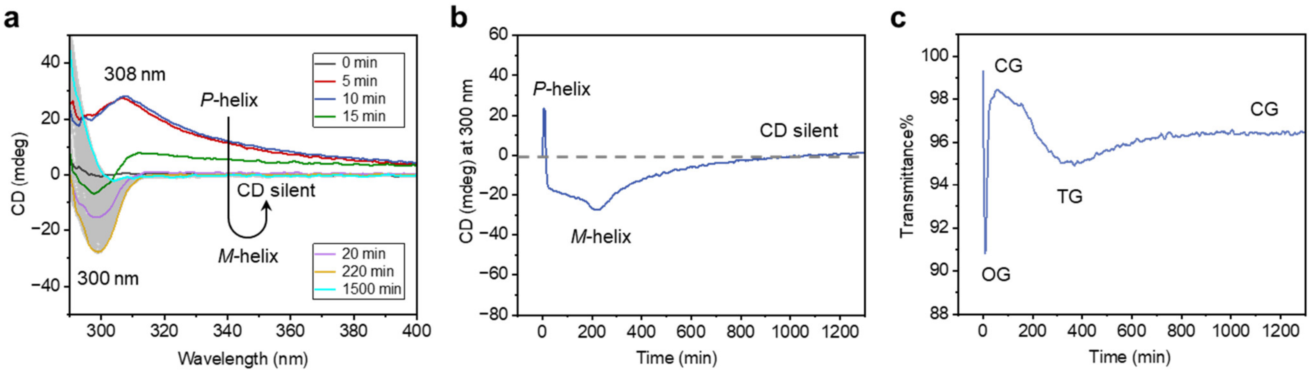

Figure S12. (a) Time-dependent CD spectra of the self-regulating gels formed by LG/4-PyB mixture, recorded during the pH evolution process. (**b**) CD signal intensity at 300 nm as a function of time. (**c**) Transmittance changes as a function of time. Conditions: 0.8% w/v LG/4-PyB (1:1) stock solution (500 μL, initial pH 5.8 ± 0.1) mixed with urea (15 μL, 2.4 M stock solution), urease (8 μL, 0.3125 g mL^{-1} stock solution), ethyl acetate (15 μL, anhydrous), esterase (10 μL, 7.5 g mL^{-1} stock solution), and KCl (3.5 μL, 1 M stock solution).

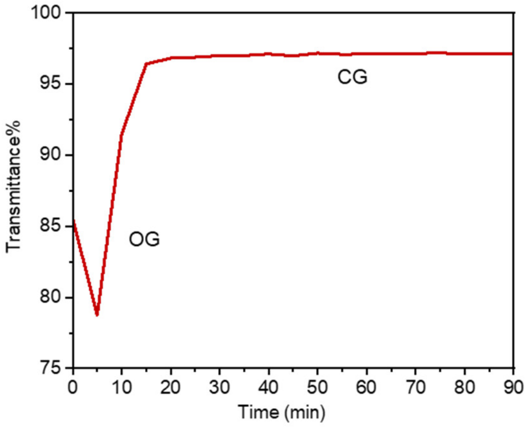

Figure S13. Time-dependent transmittance of the DG/4-PyB mixture under a unidirectional pH increase, measured at 400 nm with a 0.5 mm path length. Conditions: 0.8% w/v DG/4-PyB (1:1) stock solution (500 μL, initial pH 5.8 ± 0.1) mixed with urea (15 μL, 2.4 M stock solution), urease (8 μL, 0.3125 g mL^{-1} stock solution), and KCl (3.5 μL, 1 M stock solution).

The above time-dependent transmittance profile of the DG/4-PyB mixture illustrates the gel's dynamic structural evolution driven by a unidirectional pH increase. At the early stage, the transmittance declines to approximately 78%, indicating the formation of an opaque gel (OG). As the system evolves, a sharp rise in transmittance is observed, eventually reaching a stable value around 97%, characteristic of a uniform and fully transparent gel (CG). This gradual optical transition aligns well with the gel–gel transformation captured in Video S2 and highlights the dynamic self-assembly process of the DG/4-PyB system.

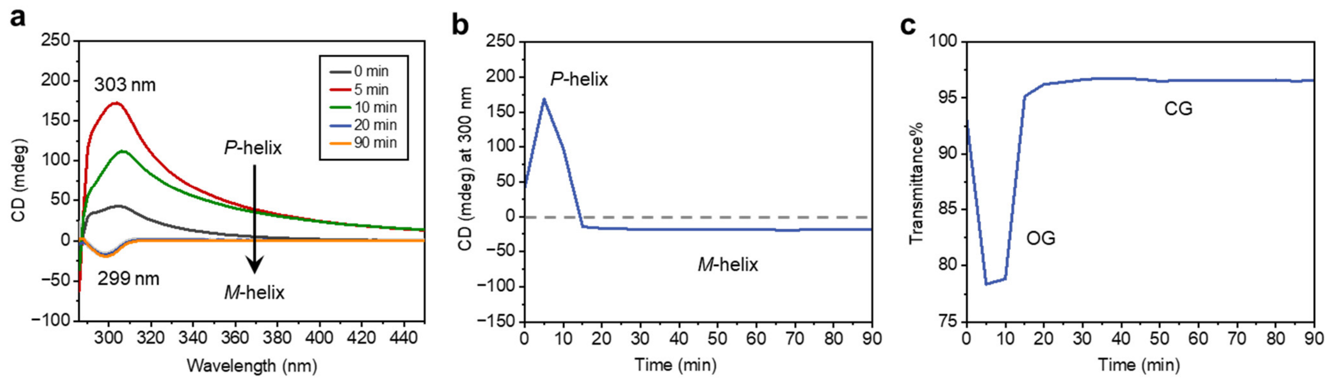

Figure S14. (a) Time-dependent CD spectra of the self-regulating gels formed by LG/4-PyB mixture, recorded during the pH evolution process. (**b**) CD signal intensity at 300 nm as a function of time. (**c**) Transmittance changes as a function of time. Conditions: 0.8% w/v DG/4-PyB (1:1) stock solution (500 μL, initial pH 5.8 ± 0.1) mixed with urea (15 μL, 2.4 M stock solution), urease (8 μL, 0.3125 g mL^{-1} stock solution), and KCl (3.5 μL, 1 M stock solution).

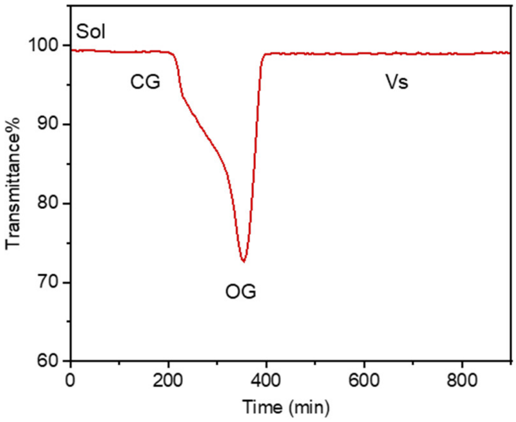

Figure S15. Time-dependent transmittance of the DG/4-PyB mixture under a unidirectional pH decrease, measured at 400 nm with a 0.5 mm path length. Conditions: 1.0% w/v DG/4-PyB (1:1) stock solution (500 μL, initial pH 10.4 ± 0.1) mixed with β-butyrolactone (10 μL).

The above time-dependent transmittance profile of the DG/4-PyB mixture illustrates a reversible structural transition of the gel system under a unidirectional pH decrease. Initially, the sample remains in a clear sol state (Sol) with a high transmittance of approximately 100%. Over time, the transmittance gradually decreases to around 95% with the formation of a clear gel (CG), followed by a further drop to a minimum of approximately 68%, indicating the transition to an opaque gel (OG). Subsequently, the transmittance rapidly increases, and the system returns to a highly transparent state (Vs), with transmittance recovering to nearly 100%. These dynamic phase changes highlight the pH-driven, time-dependent self-assembly and disassembly behavior of the DG/4-PyB system.

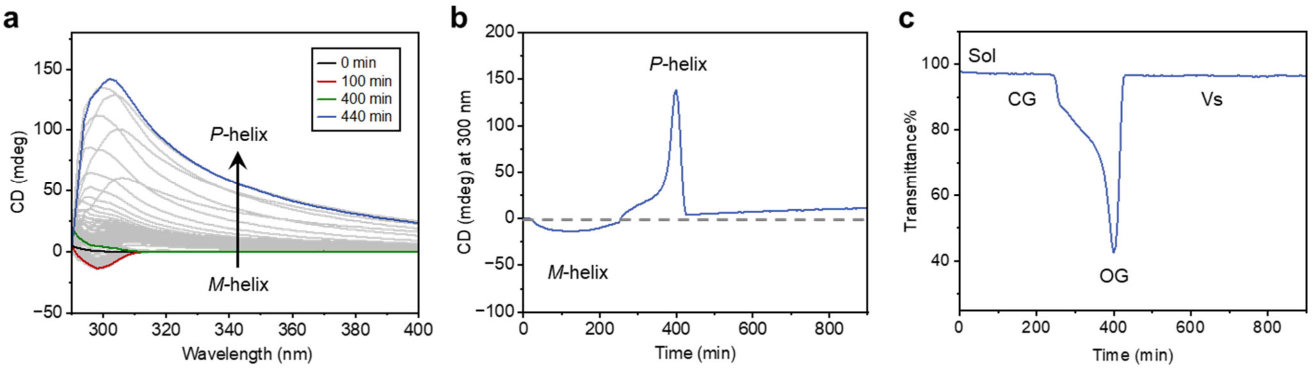

Figure S16. (a) Time-dependent CD spectra of the sample formed by LG/4-PyB mixture, recorded during the pH evolution process. (**b)** CD signal intensity at 300 nm as a function of time. (**c)** Transmittance changes as a function of time. Conditions: 1.0% w/v LG/4-PyB (1:1) stock solution (500 μL, initial pH 10.4 ± 0.1) mixed with β-butyrolactone (10 μL).

References

(1) Liptak, M. D.; Shields, G. C., Accurate pK_a Calculations for Carboxylic Acids Using Complete Basis Set and Gaussian-n Models Combined with CPCM Continuum Solvation Methods. *J. Am. Chem. Soc.* **2001,** *123* (30), 7314–7319.

(2) Jang, Y. H.; Goddard, W. A.; Noyes, K. T.; Sowers, L. C.; Hwang, S.; Chung, D. S., pKa Values of Guanine in Water: Density Functional Theory Calculations Combined with Poisson−Boltzmann Continuum−Solvation Model. *J. Phys. Chem. B* **2003,** *107* (1), 344–357.

(3) Frisch, M. J. et al. Gaussian 16 (Gaussian Inc., 2016).

(4) Raghavachari, K., Perspective on "Density functional thermochemistry. III. The role of exact exchange". *Theor. Chem. Acc.* **2000,** *103*, 361–363.

(5) Grimme, S.; Ehrlich, S.; Goerigk, L., Effect of the Damping Function in Dispersion Corrected Density Functional Theory. *J. Comput. Chem.* **2011,** *32* (7), 1456–1465.

(6) Marenich, A. V.; Cramer, C. J.; Truhlar, D. G., Universal Solvation Model Based on Solute Electron Density and on a Continuum Model of the Solvent Defined by the Bulk Dielectric Constant and Atomic Surface Tensions. *J. Phys. Chem. B* **2009,** *113* (18), 6378–6396.

(7) Lu, T.; Chen, F., Multiwfn: A multifunctional wavefunction analyzer. *J. Comput. Chem.* **2012,** *33* (5), 580–592.

(8) Wang, J.; Wolf, R. M.; Caldwell, J. W.; Kollman, P. A.; Case, D. A., Development and testing of a general amber force field. *J. Comput. Chem.* **2004,** *25* (9), 1157–1174.

(9) Tian Lu, Sobtop, Version 1.0, http://sobereva.com/soft/Sobtop (accessed on 28, 12, 2024).

(10) Van Der Spoel, D.; Lindahl, E.; Hess, B.; Groenhof, G.; Mark, A. E.; Berendsen, H. J. C., GROMACS: Fast, flexible, and free. *J. Comput. Chem.* **2005,** *26* (16), 1701–1718.

(11) Bannwarth, C.; Caldeweyher, E.; Ehlert, S.; Hansen, A.; Pracht, P.; Seibert, J.; Spicher, S.; Grimme, S., Extended tight-binding quantum chemistry methods. *WIREs Comput. Mol. Sci.* **2021,** *11* (2), e1493.

(12) Bannwarth, C.; Ehlert, S.; Grimme, S., GFN2-xTB—An Accurate and Broadly Parametrized Self-Consistent Tight-Binding Quantum Chemical Method with Multipole Electrostatics and Density-Dependent Dispersion Contributions. *J. Chem. Theory Comput.* **2019,** *15* (3), 1652–1671.

(13) Legault, C. Y. CYLview, 1.0b; Université de Sherbrooke, 2009 (http://www.cylview.org).

(14) Dreos, R.; Nardin, G.; Randaccio, L.; Siega, P.; Tauzher, G.; Vrdoljak, V., A Molecular Box Derived from Cobaloxime Units Held Together by 4-Pyridinylboronic Acid Residues. *Inorg. Chem.* **2001,** *40* (22), 5536–5540.

(15) Ortega-Valdovinos, L. R.; Chino-Cruz, J. G.; Yatsimirsky, A. K., Zwitterion–neutral form equilibria and binding selectivity of pyridineboronic acids. *Org. Biomol. Chem.* **2023,** *21* (36), 7395–7409.

(16) Ramautar, R.; Torano, J. S.; Somsen, G. W.; de Jong, G. J., Evaluation of CE methods for global metabolic profiling of urine. *Electrophoresis* **2010,** *31* (14), 2319–2327.

(17) Wu, X.; Li, Z.; Chen, X.-X.; Fossey, J. S.; James, T. D.; Jiang, Y.-B., Selective sensing of saccharides using simple boronic acids and their aggregates. *Chem. Soc. Rev.* **2013,** *42* (20), 8032–8048.

(18) Xie, X.-Q.; Zhang, Y.; Wang, M.; Liang, Y.; Cui, Y.; Li, J.; Liu, C.-S., Programmable Transient Supramolecular Chiral G-quadruplex Hydrogels by a Chemically Fueled Non-Equilibrium Self-assembly Strategy. *Angew. Chem. Int. Ed.* **2022,** *61* (9), e202114471.

(19) Peters, G. M.; Skala, L. P.; Plank, T. N.; Hyman, B. J.; Manjunatha Reddy, G. N.; Marsh, A.; Brown, S. P.; Davis, J. T., A G4·K^+ Hydrogel Stabilized by an Anion. *J. Am. Chem. Soc.* **2014,** *136* (36), 12596–12599.

(20) Peters, G. M.; Skala, L. P.; Plank, T. N.; Oh, H.; Reddy, G. N.; Marsh, A.; Brown, S. P.; Raghavan, S. R.; Davis, J. T., G4-Quartet·M^+ Borate Hydrogels. *J. Am. Chem. Soc.* **2015,** *137* (17), 5819–5827.

(21) Fidler, A. L.; Boudko, S. P.; Rokas, A.; Hudson, B. G., The triple helix of collagens – an ancient protein structure that enabled animal multicellularity and tissue evolution. *J. Cell Sci.* **2018,** *131* (7), jcs203950.
